# KISL: knowledge-injected semi-supervised learning for biological co-expression network modules

**DOI:** 10.3389/fgene.2023.1151962

**Published:** 2023-05-02

**Authors:** Gangyi Xiao, Renchu Guan, Yangkun Cao, Zhenyu Huang, Ying Xu

**Affiliations:** ^1^ College of Computer Science and Technology, Jilin University, Changchun, China; ^2^ School of Artificial Intelligence Jilin University, Changchun, China; ^3^ School of Medicine, Southern University of Science and Technology, Shenzhen, Guangdong, China

**Keywords:** biological co-expression network, factor analysis, semi-supervised learning algorithm, network modules identification, feature selection

## Abstract

The exploration of important biomarkers associated with cancer development is crucial for diagnosing cancer, designing therapeutic interventions, and predicting prognoses. The analysis of gene co-expression provides a systemic perspective on gene networks and can be a valuable tool for mining biomarkers. The main objective of co-expression network analysis is to discover highly synergistic sets of genes, and the most widely used method is weighted gene co-expression network analysis (WGCNA). With the Pearson correlation coefficient, WGCNA measures gene correlation, and uses hierarchical clustering to identify gene modules. The Pearson correlation coefficient reflects only the linear dependence between variables, and the main drawback of hierarchical clustering is that once two objects are clustered together, the process cannot be reversed. Hence, readjusting inappropriate cluster divisions is not possible. Existing co-expression network analysis methods rely on unsupervised methods that do not utilize prior biological knowledge for module delineation. Here we present a method for identification of outstanding modules in a co-expression network using a knowledge-injected semi-supervised learning approach (KISL), which utilizes apriori biological knowledge and a semi-supervised clustering method to address the issue existing in the current GCN-based clustering methods. To measure the linear and non-linear dependence between genes, we introduce a distance correlation due to the complexity of the gene-gene relationship. Eight RNA-seq datasets of cancer samples are used to validate its effectiveness. In all eight datasets, the KISL algorithm outperformed WGCNA when comparing the silhouette coefficient, Calinski-Harabasz index and Davies-Bouldin index evaluation metrics. According to the results, KISL clusters had better cluster evaluation values and better gene module aggregation. Enrichment analysis of the recognition modules demonstrated their effectiveness in discovering modular structures in biological co-expression networks. In addition, as a general method, KISL can be applied to various co-expression network analyses based on similarity metrics. Source codes for the KISL and the related scripts are available online at https://github.com/Mowonhoo/KISL.git.

## 1 Introduction

To study the functions of genes at a system level, a key is to understand how genes work together. A basic assumption is that co-expressed genes tend to work in the same subsystem. Co-expression networks (GCN) (Yip and Horvath, 2007a) are commonly used to describe such subsystems based on statistical correlations among the expressions of the relevant genes. Typically, each node in such an undirected network represents a distinct gene and a weighted edge between two nodes denotes the two genes with correlated expressions while the edge weight represents the correlation level.

One goal when studying such a network is to discover densely connected subnetworks, also referred to as functional modules or clusters, as co-expressed genes tend to be transcriptionally coregulated. WGCNA (Zhang and Horvath, 2005) is a most widely used software for GCN construction, and can be used to identify modules of highly co-expressed genes. Briefly, WGCNA constructs a weighted co-expression network based on the Pearson correlation coefficients among provided gene expressions; uses a topological overlap structure measure (TOM) (Ravasz et al., 2002) of nodes to identify modules; and utilizes eigengene and intramodule hub genes to summarize such modules (Langfelder and Horvath, 2008). WGCNA identifies gene modules by using hierarchical clustering, giving rise to a tree-like structure. The advantage of the hierarchical clustering method is its simplicity, but the process for generating a hierarchical clustering tree is irreversible.

Multiple developments have been made aiming to improve the TOM measure. Among them, Li et al. proposed a bottom-up multi-node topological overlap measure (MTOM) that selects nodes with the highest neighborhood size to form modules based on multiple nodes. (Yip and Horvath, 2007b) developed a generalized topological overlap measure, called GTOM. Compared to TOM that considers only the nodes directly adjacent to the target gene pair, GTOM considers neighboring nodes that are within K steps away from the target gene pair, where K is a parameter to be selected by the user. Thus, GTOM is more sensitive to higher-order connections. Hou et al. (2021) introduced the K-means method to WGCNA to add additional steps to improve the module-identification results of WGCNA. A few other algorithms have been deployed to analyze gene co-expression networks, such as the flow simulation-based module discovery method (MCL) (Hwang et al., 2006), the graph partitioning-based method (Qcut) (Ruan and Zhang, 2008), and the density model-based method (MCODE) (Bader and Hogue, 2003).

One common issue with all these methods is: they use only unsupervised methods for clustering or module identification, but do not make effective use of prior biological knowledge. In addition, WGCNA uses hierarchical clustering to identify gene modules. One drawback of hierarchical clustering is that once two objects are clustered together, the process cannot be reversed. Therefore, regrouping of inappropriately clustered items is not doable. Analyses of the improved methods of WGCNA for refining its module identification results shows that the methods could not solve the problem of generating an unreasonable number of clusters. The purpose of this paper is to develop an effective method for module identification in a co-expression network to improve the of these two issues in existing methods.

Here we present a method for identification of outstanding modules in a co-expression network using a knowledge-injected semi-supervised learning approach (KISL), which utilizes *apriori* biological knowledge and a semi-supervised clustering (Basu et al., 2004) method to address the issue existing in the current GCN-based clustering methods. A comparative analysis of our algorithm with the WGCNA method on eight human cancer datasets has revealed the effectiveness of our algorithm in discovering modular structures in co-expression networks, paving the way for more accurate and useful GCN analysis.

## 2 Methods

### 2.1 WGCNA and KISL algorithms

**FIGURE 1:**
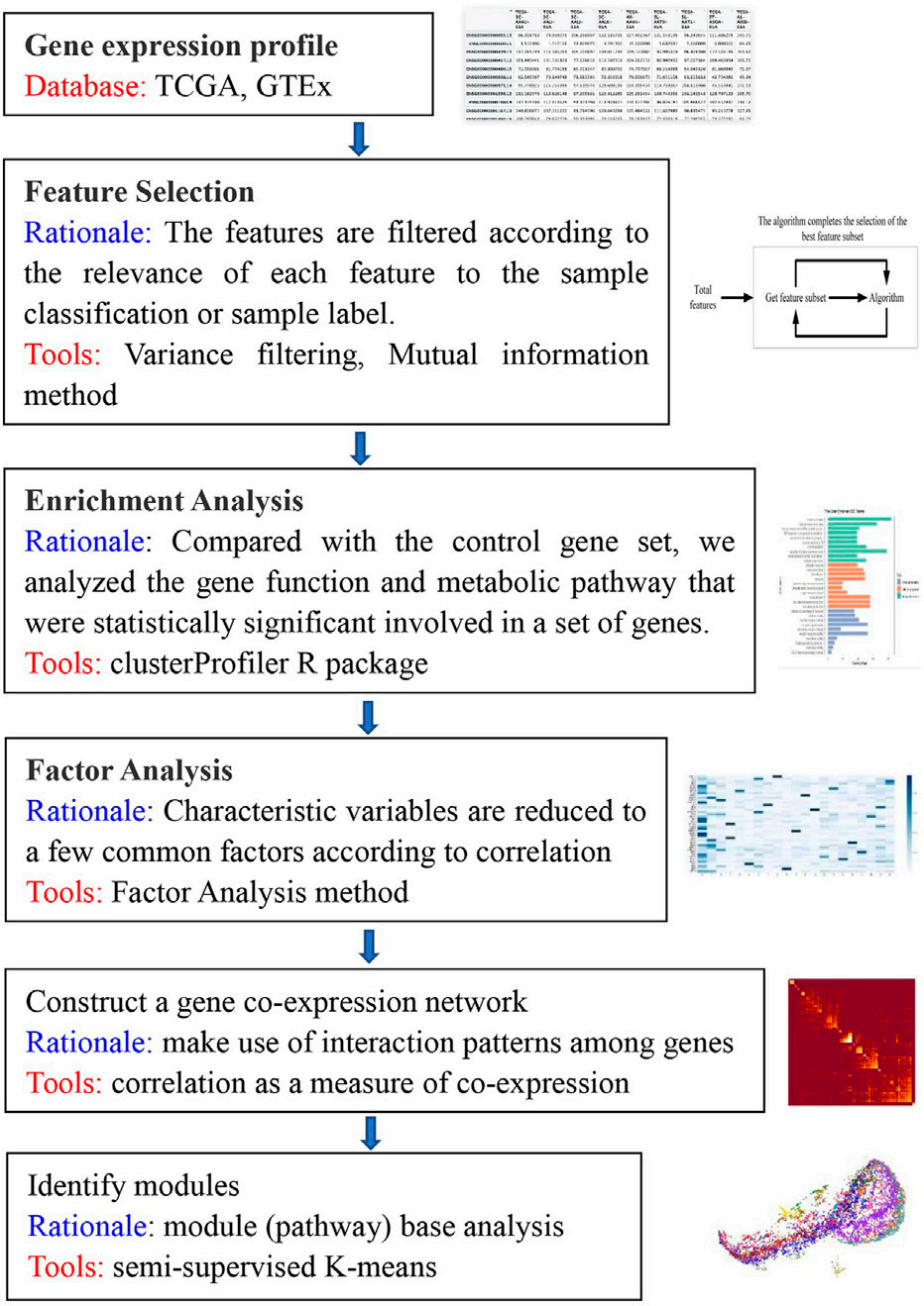
Overview of the algorithm. This flowchart briefly describes the main. steps of the KISL algorithm.

We sought to identify modules consisting of highly functionally related genes. The structure of our algorithm is shown in Figure 1, consisting of three main stages. The first stage covers data preprocessing, variance analysis and feature selection to generate a gene expression profile matrix. The second stage is to construct clustering constraints by using factor analysis, to perform Gene Ontology (GO) enrichment analysis, and to perform factor analysis based on gene expression profiles for the set of genes covered by enriched GO/BP pathways. The result is a factor-loading matrix. The factor coefficients are binarized through thresholding, a subset of genes affected by a single factor is screened to form the “must-link” gene clusters, and all gene clusters from the pathway screening together form the *apriori* constraints for module identification in the co-expression network. The third stage is to construct the GCN and then use a semi-supervised algorithm in combination with the *apriori* constraints for identification of the GCN functional modules.

The inputs to the semi-supervised algorithm are the GCN network, the *apriori* constraints and the number of clusters k (the value of k is set according to the learning curve by the user given a value interval for k). The main purpose of the algorithm is to calculate the connectivity of genes to the module mean vector in each module and to assign genes to the modules that are most highly connected to them. Here, the mean vector $\mu_{j}$ of module j is defined as in Eq. (1).

$$\mu_{j}=\frac{1}{\left|C_{j}\right|}\sum_{x\epsilon C_{j}}x_{i}$$ (1)

where $x_{i}$ is the expression profile of gene i , $C_{j}$ is the set of all genes in module j , and $\left|C_{j}\right|$ denotes the number of genes in module j . We calculate the distance $d_{ij}={\left\|x_{i}-\mu_{j}\right\|}_{2}$ between the sample $x_{i}$ and each mean vector $\mu_{j}\left(1\leq j\leq k\right)$ . We count ${count}_{j}\left(j=1,2,\ldots ,k\right)$ of other samples in the constraint set containing sample $x_{i}$ in each clustering cluster. The distance $d_{ij}=d_{ij}+{count}_{j}$ between sample $x_{i}$ and module j is adjusted according to the constraint. For each gene i we set its module label to the label of the mean vector that minimizes $d_{ij}$ . We then recalculate the mean vector of genes in each module and repeat the previous steps until no cluster assignment changes or the preset maximum number of iterations is reached. Additionally, tool KISL includes several additional functions designed to aid the user in visualizing input data and results. These functions rely on basic plotting functions provided in python and the R packages WGCNA (Langfelder and Horvath, 2008). The code of the KISL algorithm are available online at https://github.com/Mowonhoo/KISL.git.

### 2.2 Construction of the gene co-expression network

Measuring the co-expression relationship between genes is a key issue in the construction of gene co-expression networks. However, commonly used correlation measures, including linear (e.g., Pearson correlation) and monotonic (e.g., Spearman correlation) dependence measures, are not sufficient to observe the nature of real biological systems. Szekely et al. (Székely et al., 2007; Székely and Rizzo, 2009) proposed distance correlation for both linear and non-linear dependencies. Distance correlation reveals more about the complex biological relationships between gene profiles than other correlation metrics, which helps to provide more meaningful modules in the analysis of gene co-expression networks. However, the time complexity associated with computing the distance is high and requires more computational resources (Hou et al., 2022). However, for biological analysis we seek higher reliability and completeness of information mining, therefore, in this study, we use distance correlation to measure the relationship between genes. To optimize the time spent by the algorithm, the features can be optionally downscaled by using the principal component analysis (PCA) method before calculating the correlation coefficients between genes, and feature retention is filtered by setting a threshold based on the PCA variance interpretation rate.

The distance correlation coefficient can reveal an arbitrary relationship between the variables. When the Pearson correlation coefficient is 0, we cannot determine whether the two variables are independent, but if the distance correlation coefficient is 0, then we can conclude that the two variables are independent of each other (Pearson and Galton, 1895; Székely et al., 2007; Székely and Rizzo, 2009). The distance correlation coefficient of two variables u and v is denoted as $\hat{d}corr\left(u,v\right)$ . When $\hat{d}corr\left(u,v\right)=0$ , the two variables are independent of each other. The larger $\hat{d}corr\left(u,v\right)$ is, the stronger the correlation between u and v . Let the random sample of the overall $\left(u,v\right)$ be $\left\{\left(u,v\right),i=1,2,\ldots ,n\right\}$ and Szekely et al. (Székely et al., 2007; Székely and Rizzo, 2009) defined the sample estimate of the distance correlation coefficient between two random variables u and v as Eq. 2.

$$\hat{d}corr\left(u,v\right)=\frac{\hat{d}cov\left(u,v\right)}{\sqrt{\hat{d}cov\left(u,u\right)\hat{d}cov\left(v,v\right)}}$$ (2)

where $\hat{d}{cov}^{2}\left(u,v\right)=\hat{S_{1}}+\hat{S_{2}}-2\hat{S_{3}}$ , $\hat{S_{1}}$ , $\hat{S_{2}}$ and $\hat{S_{3}}$ are shown in Eqs 3, 4, 5, respectively.

$$\hat{S_{1}}=\frac{1}{n^{2}}\sum_{i=1}^{n}\sum_{j=1}^{n}{\left\|u_{i}-u_{j}\right\|}_{d_{u}}{\left\|v_{i}-v_{j}\right\|}_{d_{v}}$$ (3)

$$\hat{S_{2}}=\frac{1}{n^{2}}\sum_{i=1}^{n}\sum_{j=1}^{n}{\left\|u_{i}-u_{j}\right\|}_{d_{u}}\frac{1}{n^{2}}\sum_{i=1}^{n}\sum_{j=1}^{n}{\left\|v_{i}-v_{j}\right\|}_{d_{v}}$$ (4)

$$\hat{S_{3}}=\frac{1}{n^{3}}\sum_{i=1}^{n}\sum_{j=1}^{n}\sum_{l=1}^{n}{\left\|u_{i}-u_{l}\right\|}_{d_{u}}{\left\|v_{i}-v_{l}\right\|}_{d_{v}}$$ (5)

Similarly, $\hat{d}cov\left(u,u\right)$ and $\hat{d}cov\left(v,v\right)$ can be calculated.

The gene adjacency matrix is obtained by power-lawing the gene correlation matrix with a “soft” threshold power , and then the TOM of the adjacency network is calculated to construct the gene co-expression network. The construction of gene co-expression networks based on the TOM metric has been shown to have better results than direct module identification based on the adjacency graph (Langfelder et al., 2008).

We have kept the Pearson correlation coefficient for measuring the interrelationship between genes among the optional parameters of the functional function used to construct the co-expression network in order to increase the applicability and scalability of our algorithm and to meet the various needs of users. We have also given the mutual information method (MI) as an optional parameter, so that users can choose the parameters according to their needs. A MI measures the entropy of gene interactions to evaluate their relationship. In comparing linear and non-linear methods for measuring gene dependence, Zhang et al. found that the mutual information method combined linear and non-linear interactions has some advantages over linear or non-linear methods (Jiang and Zhang, 2022). Moreover, the MI between two variables is symmetric, which means that MI-based methods infer undirected interactions (Jia and Zhang, 2022). Additionally, we simulated and generated 10 pairs of high-dimensional variables with different dependencies, and then used them to measure the relationship between these variable pairs in order to compare the characteristics of distance correlation, mutual information, and Pearson correlation coefficient to capture the complex relationship between variables. Calculations are performed using Python packages sklearn (Pedregosa et al., 2011), dcor (Ramos-Carreño and Torrecilla, 2022), and scipy (Virtanen et al., 2020). The supplementary Material 6 (Supplementary Figure S1) contains the pertinent results.

### 2.3 Topological characteristics of GCN

Network topology analysis is an important tool for understanding network characteristics at the system level. Network centrality analysis and global network topology analysis are two levels used to analyze the network from the system level. A key concept in network analysis is node connectivity (centrality). A central node (called a hub) is a node that is densely connected to other nodes. Co-expression networks have global topological properties of scale-free distributions, functional modular networks, and small-world properties. For weighted networks, Zhang and Horvath et al. (Zhang and Horvath, 2005) also defined the corresponding connectivity, intramodule connectivity metric and generalized scale-free topology for weighted networks.

1) Connectivity in weighted networks

The connectivity metric based on the weighted adjacency network is defined as Eq. 6.

$$W_{i}=\sum_{j=1}^{n}w_{ij}$$ (6)

where $w_{ij}$ is the adjacency between two nodes i and j . Thus, if a node has high adjacency with many other nodes, then it has high connectivity $W_{i}$ based on the weighted adjacency network.

A network connectivity metric is defined for a specific module’s genes (intramodule connectivity). The intramodule connectivity (unweighted network node connectivity also commonly referred to as “degree”) of gene i within module q is calculated as in Eq. 7.

$$\left.{within(k}_{i}^{(q)})=\underset{j}{\sum }w_{ij}\;(j=1,2,\ldots ,n\left(q\right)\right)$$ (7)

where $n\left(q\right)$ denotes the number of genes within module q .

2) Module density

The dense connectivity property between genes within module q can be measured by the average neighboring degree of module genes, defined as the module density, as shown in Eq. 8.

$$Density\left(A^{\left(q\right)}\right)=\frac{\sum_{i}\sum_{j\neq i}w_{ij}^{\left(q\right)}}{n^{\left(q\right)}\left(n^{\left(q\right)}-1\right)}$$ (8)

where $A^{\left(q\right)}$ denotes the $n^{\left(q\right)}\times n^{\left(q\right)}$ adjacency matrix corresponding to the subnetwork formed by the genes of module q .

3) Generalized scale-free topology

The frequency distribution $p\left(k\right)$ of node connectivity in a gene neighborhood network follows the power law $p\left(k\right)∼k^{-\gamma }$ . where k is the node connectivity (Langfelder et al., 2008). The square of the correlation between $\log_{10}p\left(k\right)$ and $\log_{10}k$ can be used to measure the degree to which the network satisfies the scale-free topology, i.e., the model fit index $R^{2}$ for a linear model regressing $\log_{10}p\left(k\right)$ on $\log_{10}k$ . If the $R^{2}$ value is close to 1, there is a linear relationship between $\log_{10}p\left(k\right)$ and $\log_{10}k$ .

### 2.4 Construction methods for *a priori* constraints

Thanks to the results of work in related fields of research it has been possible to obtain many biological explanations of the relationships between genes. The Gene Ontology (GO) database is one of the common gene annotation systems used in bioinformatics research, and it defines a structured standard biological model that allows the description of gene and protein functions in various organisms in terms of cellular components, biological processes and molecular functions.

The enrichment analysis enables the annotation and classification of genes to obtain a subset of genes grouped according to different gene functions, and the annotated results can be transformed to constitute *a priori* constraints for module identification algorithms to improve the modular biological interpretation of functional module identification of co-expression networks. We introduced factor analysis (Swisher et al., 2004; Ferrando, 2021), a statistical method for extracting common factors from groups of variables, to construct intergenic correlation constraints. The British psychologist C.E. Spearman first proposed it. Factor analysis can identify the common influences embedded in multiple variables. By grouping variables of the same nature into a common factor, the number of variables can be reduced. as shown in Eq. 9 below.

$$\left\{\begin{array}{c} X_{1}=a_{11}F_{1}+a_{12}F_{2}+\ldots +a_{1m}F_{m}+\epsilon_{1}\\ X_{2}=a_{21}F_{1}+a_{22}F_{2}+\ldots +a_{2m}F_{m}+\epsilon_{2}\\ \cdots \\ X_{P}=a_{p1}F_{1}+a_{p2}F_{2}+\ldots +a_{pm}F_{m}+\epsilon_{p} \end{array}\right.$$ (9)

where F denotes the common factor, X denotes the original variable, and ϵ denotes the part of the original variable that cannot be represented by the common factor. The number of original variables is generally satisfied as greater than or equal to the number of factors (i.e., m≤p ). The factors F are independent of each other and have a variance of 1. The correlation between the common factor and ϵ is 0 and the correlation between ϵ is 0.

Before performing factor analysis, the Kaiser-Meyer-Olkin test (KMO test) and Bartlett’s test of sphericity were performed on the features to determine whether the gene expression profile was suitable for factor analysis. Then, by calculating the eigenvalues of the gene correlation matrix and ranking them, the common factors with eigenvalues greater than 1 were extracted according to Kaiser’s principle, and the cumulative total variance contribution rate was ensured to be greater than 0.85 according to the variance contribution rate accumulation principle. This process ensures that the extracted common factors cover enough information contained in the original gene expression profile and better replace the original gene characteristics. The factor loading coefficients are then derived and transformed by orthogonal rotation of the loading coefficients to obtain the factor loading matrix and then to analyze the characteristics of the factor coefficients for each gene. The factor loading coefficient matrix is then binarized to filter out the subset of genes that depend on a certain common factor in the same pathway, and these genes are only highest correlated with this main factor. The constrained gene set is obtained by performing factor analysis on all GO terms enriched in the gene expression profile and then by merging the subsets with common overlapping genes.

### 2.5 Clustering evaluation metrics

The silhouette coefficient (RousseeuwSilhouettes, 1987), the Calinski-Harabasz index (Caliński and Harabasz, 1974) and the Davies-Bouldin index (Davies and Bouldin, 1979) are common and valid internal measures to evaluate the validity of clustering. The silhouette coefficient is a measure of how similar an observation is to its own cluster compared to other clusters, and it takes values from −1 to 1. A value of 1 indicates that the clusters are far from each other and clearly distinguished, a value of 0 indicates that the distance between clusters is non-significant, and a value of −1 indicates that the clusters are incorrectly assigned. The Calinski-Harabasz index is also known as the variance ratio criterion. For cluster q , the Calinski-Harabasz index is given by the ratio of the between-cluster dispersion mean to the within-cluster dispersion, and a higher Calinski-Harabaz index indicates better clustering. The physical meaning of the Davies-Bouldin index is the ratio of the sum of the mean sample distance (i.e., intracluster sample distance) of each cluster to the distance between the centroids of the two clusters (i.e., intercluster sample distance); given two clusters, the smaller the value is, the better.

### 2.6 Gene function annotation tools

The database for annotation, visualization and integrated discovery (DAVID) provides researchers with a comprehensive set of functional annotation tools to understand the biological significance behind large lists of genes (Huang et al., 2009). DAVID integrates biological data and analysis tools to provide systematic, integrated biofunctional annotation information for large-scale gene and protein lists to help users extract biological information. Here, we used the rich scores from the DAVID functional annotation clustering tool—the geometric mean (logarithmic scale) of the *p* values of the members of the corresponding annotation clusters for ranking their biological significance. The clusterProfiler R package was used to obtain the Gene Ontology terms of all differentially expressed genes (Yu et al., 2012).

### 2.7 Datasets

The tumor sample dataset used in this experiment was obtained from The Cancer Genome Atlas (TCGA, http://cancergenome.nih.gov/) database, including BLCA (bladder urothelial carcinoma), BRCA (breast invasive carcinoma), COAD (colon adenocarcinoma), KIRC (kidney renal clear cell carcinoma), LUAD (lung adenocarcinoma), LUSC (lung squamous cell carcinoma), PAAD (pancreatic adenocarcinoma) and STAD (stomach adenocarcinoma) RNA-Seq data for eight tumors, and normal samples for each tumor were obtained from the Genotype-Tissue Expression (GTEx) database. The GTEx project aims to establish a repository of samples and data for studying the relationships between genetic variants, gene expression and other molecular phenotypes in a wide range of human tissues (GTEx Consortium, 2013; GTEx Consortium, 2015). First, the eight cancer datasets obtained from TCGA and GTEx databases were analyzed for differences by using the R package DESeq2 (Gentleman et al., 2004; Love et al., 2014). We set the screening criteria for differential genes as $padj<0.05,\left|\log_{2}FoldChange\right|>1$ , followed by variance filtering to screen out genes with variance less than or equal to 0, i.e., consistent expression activity on all samples. The selection of features is then done using the mutual information method. The sample type is the phenotype (clinical trait) that we employ for gene screening. After feature selection filtering, the final retained samples and gene counts are provided in Supplementary Material 1 (Supplementary Table S1). Source codes for the KISL and the related scripts are available online at https://github.com/Mowonhoo/KISL.git. The datasets from Gene expression RNA-seq were performed using TCGA: https://www.cancer.gov/tcga.

## 3 Results and DISCUSSION

### 3.1 Effect of distance correlation on various datasets

**FIGURE 2:**
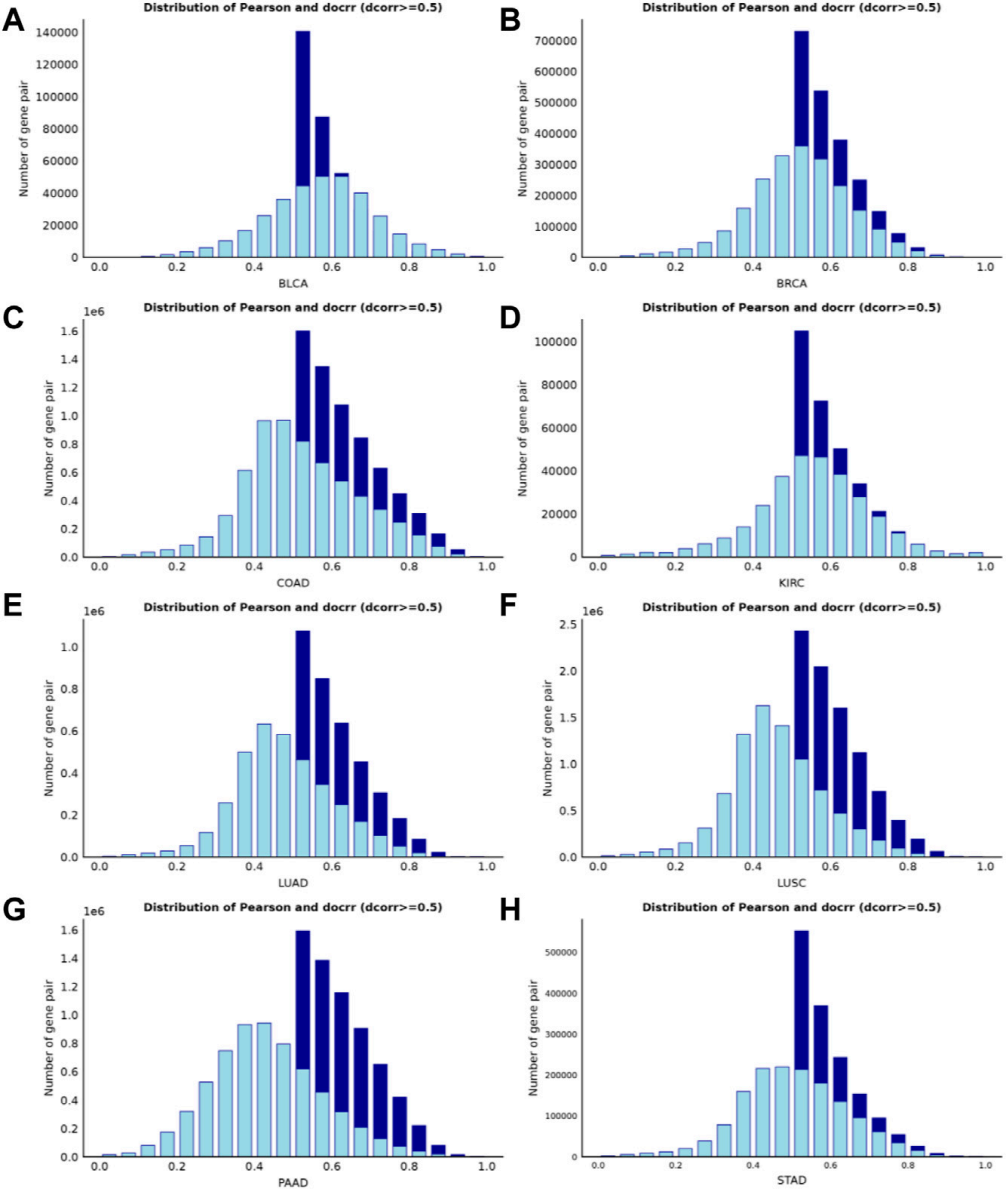
Histogram of correlation coefficients for interactions with high distance correlation scores (>0.5). The bright blue borders in each panel represents the Pearson correlations, and the dark blue borders represents the distance correlations. When using the criterion that the Pearson correlation coefficient must be greater than 0.5, more than 50% of the complex correlated data information on four of the datasets (Figures 2E–H) would be lost.

(Székely and Rizzo, 2009) verified that the value of the distance correlation is always smaller than the absolute value of the Pearson correlation for bivariate normal data. Therefore, if the distance correlation coefficient between two random variables is greater than the Pearson correlation coefficient then a complex relationship exists between them - non-binary normal data and non-linear nonmonotonic relationship. In general, correlation values greater than 0.8 are described as strong correlation, while values less than 0.5 are described as weak correlation (Castro Sotos et al., 2009). To measure the proportion of complex relationships in the dataset, we selected gene pairs with distance correlation coefficients greater than 0.5 from eight datasets. Next, we analyzed the distribution of Pearson correlation coefficients for the retained gene pairs. In the PAAD dataset, 70.88% of the gene pairs had Pearson correlation coefficients less than 0.5 (Figure 2G). In addition, the ratios in the LUSC dataset (Figure 2F), LUAD dataset (Figure 2E) and STAD dataset (Figure 2H) were 66.37%, 61.04% and 50.62%, respectively, as shown in Supplementary Material 2 (Supplementary Table S2). Both our algorithm and the standard WGCNA method use a ‘soft’ threshold power in the construction of the GCN, which amplifies the difference between strong and weak correlations. When using Pearson correlation coefficients, gene pairs with complex relationships have small correlation coefficient values, and the presence of the soft threshold further leads to a smaller weight of the two genes and increases the error, making the clustering results inaccurate.

**FIGURE 3:**
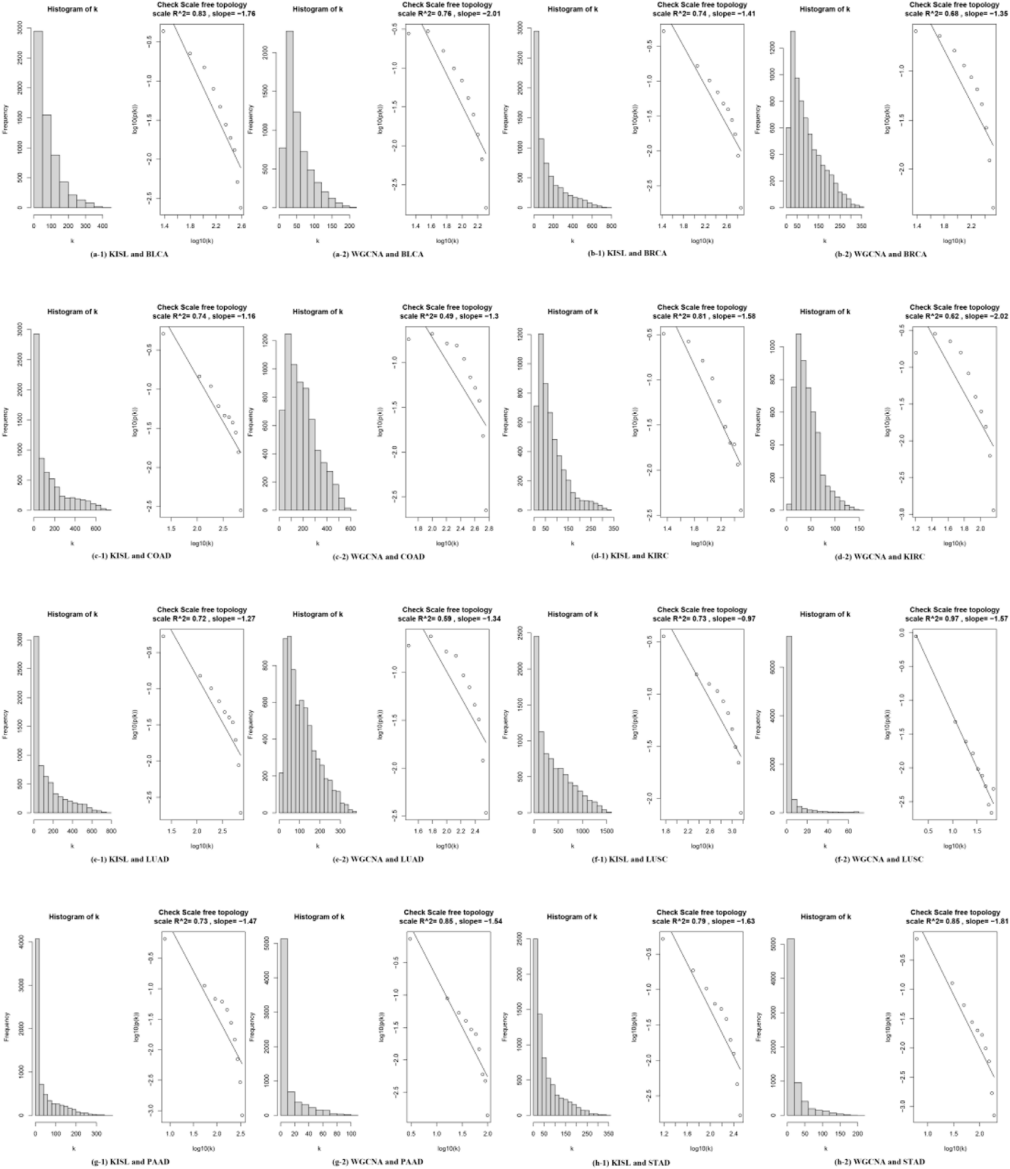
shows the scale-free topological properties of the co-expression network. The left panel shows the histogram of the network connectivity, and the right panel shows the logarithmic plot of the corresponding histogram. The approximate linear relationship (high R^2 values) represents the approximate scale-free topology. The scale-free topology is at least approximately satisfied when a suitable “soft” threshold is chosen to define the adjacency matrix for the eight selected real datasets.

It has been reported that biological networks show scale-free topology (STF) (Langfelder and Horvath, 2008; Barabási et al., 2011). It is important in SFT networks to identify the dominant hub nodes because they usually have significant influence on the network. In the case of biological networks it may mean that the genes, proteins or metabolites represented by these nodes are biologically important (Albert, 2005; Andrecut et al., 2008; Nafis et al., 2015; Atiia et al., 2020). Therefore, we investigate the SFT of the two correlation coefficients for the eight datasets. The closer the SFT fit index is to 1, the better. In Figure 3 the left panel shows the histogram of network connectivity and the right panel shows the logarithmic plot of the corresponding histogram. The approximate linear relationship (high $R^{2}$ values) indicates the approximate scale-free topology. We find that for eight datasets, both Pearson correlation coefficients and distance correlation coefficients achieve SFT when a suitable “soft” threshold power is chosen to define the adjacency matrix, and in five of them (Figures 3A–E), distance correlation shows an advantage in the scale-free fit index.

### 3.2 Constructing clustering constraints

**FIGURE 4:**
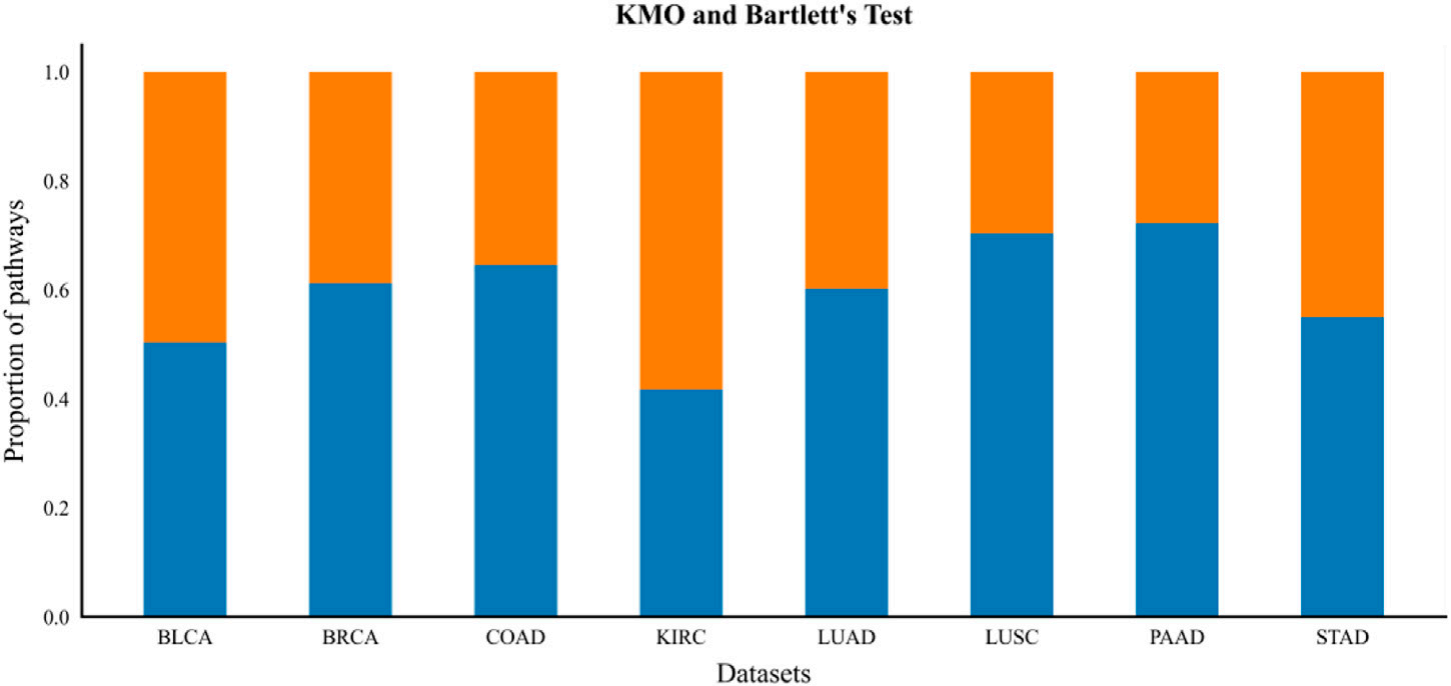
KMO and Bartlett’s test. The blue bars below the figure indicate the proportion of gene expression profiles of GO Term suitable for factor analysis after the KMO test and Bartlett‘s test of sphericity.

The KMO test and Bartlett’s test of Sphericity were used to determine whether a gene expression profile was suitable for factor analysis before all GO terms enriched in the gene expression profile were subjected to factor analysis. In this paper, the number of contained genes is greater than 5, the threshold value set by KMO test is greater than 0.6, and the *p*-value of Bartlett’s test of sphericity is set to less than 0.05 (*p*-value is less than the significance level value of 0.05, indicating a high correlation between genes in the expression profile data) of GO term for factor analysis to construct constrained gene sets. From Figure 4, we can see that the percentage of GO terms enriched in each gene expression profile data that were evaluated to be suitable for factor analysis ranged from approximately 40%–72%, which indicates that we can effectively extract *a priori* biological knowledge by introducing factor analysis methods. The factor loading matrix is binarized by setting an appropriate factor screening threshold (we set the threshold to 0.2, then each gene factor coefficient greater than 0.2 is set to 1, and less than that is set to 0). Finally, the set of constrained genes that significantly depend on a single common factor in the same pathway is obtained from the binarized factor loading matrix. All subsets of genes in all GO terms that depend on a single principal factor are filtered out, and the subsets with common overlapping genes are merged to obtain the constrained gene set. According to the clustering constraint construction process described above, the final constrained gene sets based on *a priori* biological knowledge are obtained on each dataset, and the constrained gene sets are summarized as shown in (Supplementary Table S3).

### 3.3 Evaluation based on internal metrics of clustering algorithms

**FIGURE 5:**
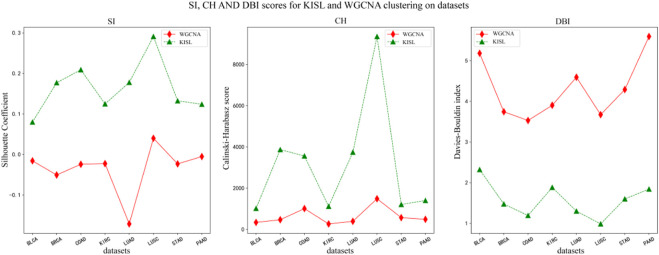
Silhouette coefficient, Calinski‒Harabasz score and Davies‒Bouldin index for the WGCNA and KISL algorithms. The evaluation value obtained by the KISL algorithm was the best in all the datasets.

**FIGURE 6:**
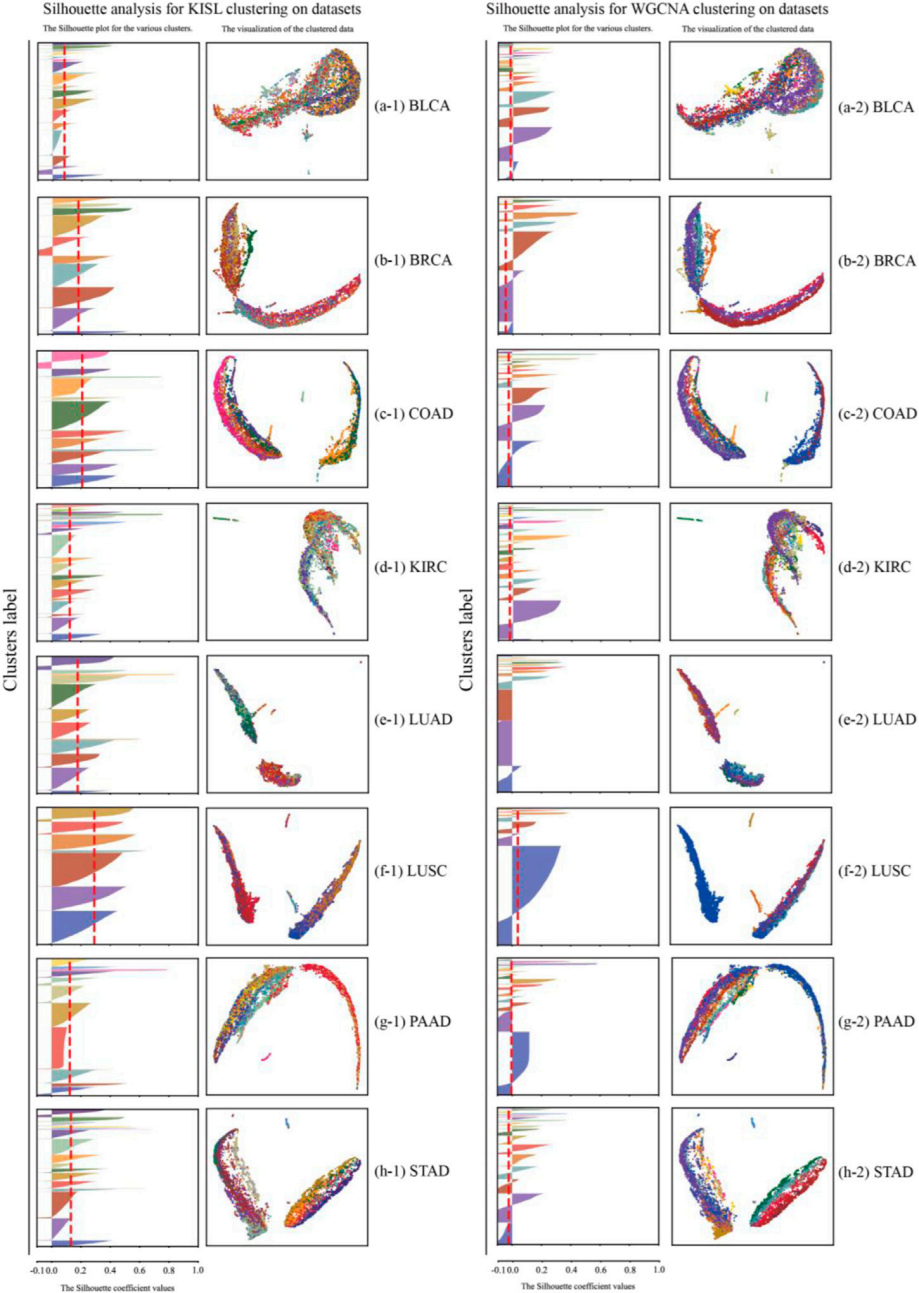
Silhouette coefficient analysis for the WGCNA and KISL algorithms. The left panel shows the results of silhouette coefficient analysis of the clusters obtained by the KISL. The right panel shows the results obtained by the base method WGCNA on the corresponding dataset. The evaluation value (the red dashed line) obtained by the KISL algorithm was the highest in all the datasets. In each panel, the left part represents the silhouette coefficient value of each sample, the *y*-axis represents the sample sequence, and the *x*-axis represents the silhouette coefficient size. UMAP visualization results are displayed on the right side of each panel.

In this section, we use the silhouette coefficient, the Calinski-Harabasz index and the Davies-Bouldin index to evaluate the quality of the WGCNA and KISL clustering results. As shown in Figure 5, the KISL algorithm obtained the highest silhouette coefficient and Calinski-Harabasz index evaluation values in all eight datasets, while obtaining the lowest Davies-Bouldin index evaluation value. Taking the silhouette coefficient evaluation metric as an example, three of the datasets, COAD, LUAD, and LUSC, obtained a boost of more than 0.3 on the dataset, and two datasets, BRCA and STAD, obtained a boost of more than 0.15 with the smallest evaluation value on the BLCA dataset but also slightly improved. It is also important to note that the silhouette coefficient value obtained by the base method is negative on most of the datasets, especially on the LUAD dataset, where it is the worst and even reaches −0.17, which means that many sample points are assigned to the wrong cluster. Our algorithm also obtained the best evaluation values for both the Calinski-Harabasz index and Davies-Bouldin index evaluation metrics. The clusters obtained by KISL have better clustering evaluation values and better aggregation of the obtained gene modules. The details of the three evaluation values of the clusters are shown in (Supplementary Table S4). In Figure 6, we plot the results of the silhouette coefficient analysis for the KISL algorithm (the left side) and the Pearson-based WGCNA (the right side) corresponding to the eight datasets. The closer the silhouette coefficient to 1, the better the clustering result. The evaluation value obtained by the KISL algorithm was the highest in all the datasets.

### 3.4 Analysis of the nature of the recognition module

**FIGURE 7:**
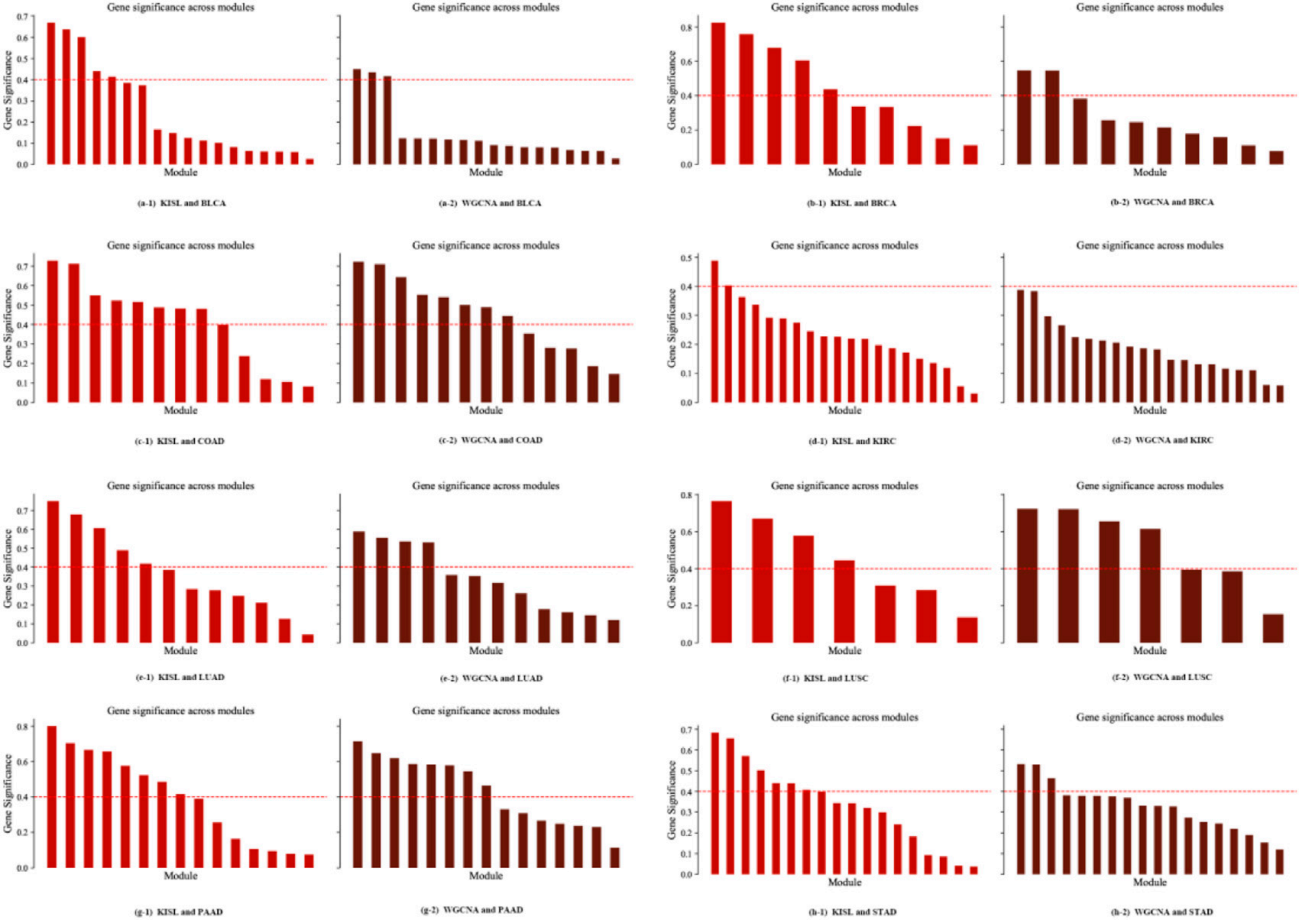
Module significance metric. The gene module significance threshold is set to 0.4 (the red dashed line), and our algorithm obtains more high gene significance modules on five of the datasets, BLCA, BRCA, KIRC, LUAD and STAD (Figures 7A,B,D,E,H), while on the other three datasets our method obtains the same number of high significance modules as the base method.

The module significance measure was defined as the average gene significance of all genes in the module. We used absolute values to define the relevance-based gene module significance metric. The results of the significance of each module identified on the eight datasets are shown in Figure 7. We use a gene module significance of 0.4 (the red dashed line) as the threshold, and we find that our algorithm obtains more high gene significance modules on five of the datasets BLCA, BRCA, KIRC, LUAD, and STAD (Figures 7A, B, D, E, H), while on the other three datasets our method obtains the same number of high significance modules as the base method.

**FIGURE 8:**
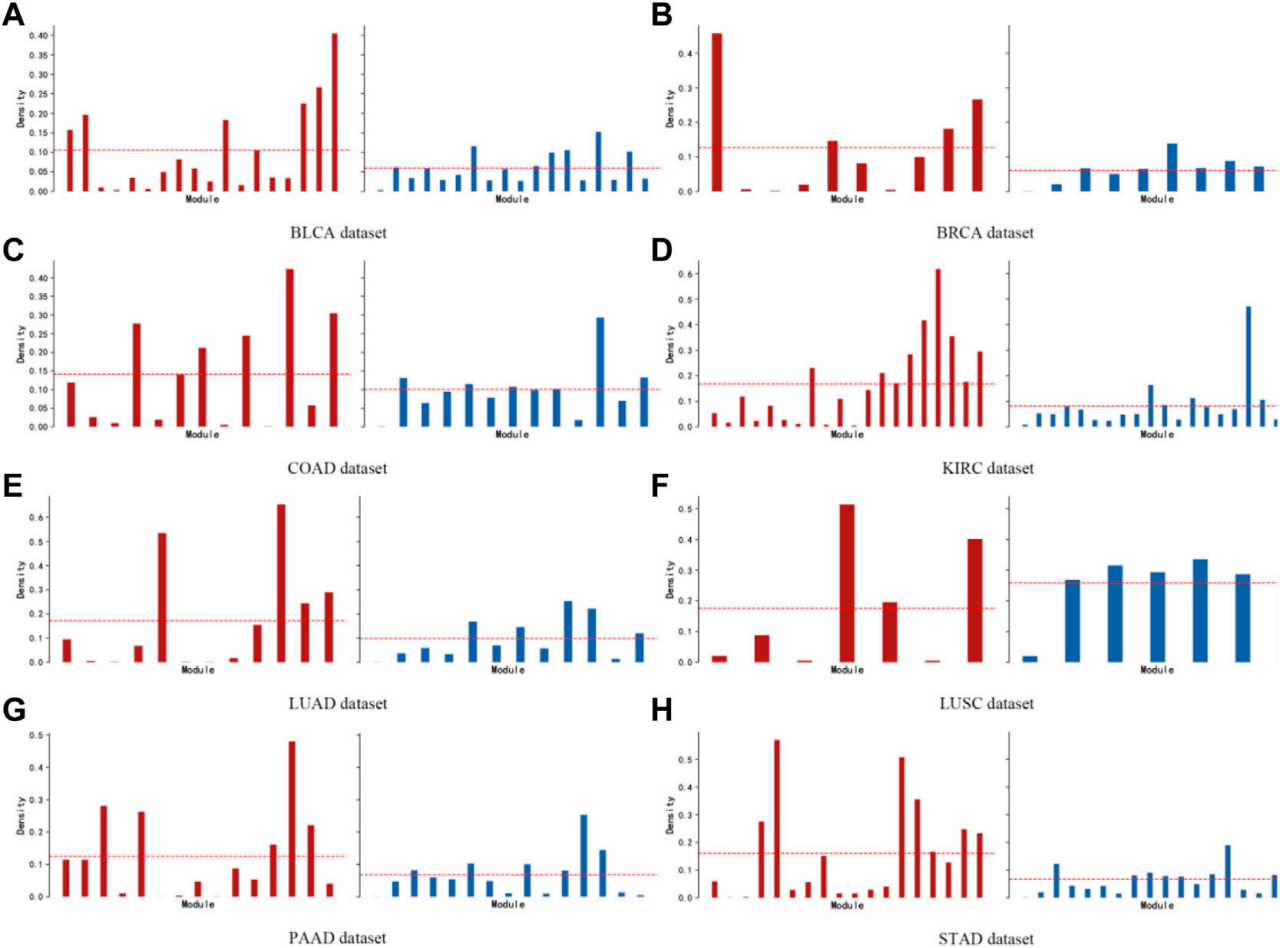
Module density. The brown bar plot indicates the module density obtained by the KISL algorithm on each dataset, and the blue bar plot indicates the results of the widely used method WGCNA. Our method obtains a larger average module density on seven of the datasets and has a larger number of modules with a larger density. In addition, the top 3 modules with the highest module density on all eight datasets are obtained by our algorithm.

A network connectivity metric is defined for module-specific genes (intramodule connectivity). The intramodular connectivity of genes within a module is calculated, and the dense connectivity property between genes within a module is measured by the average adjacency of the module genes, defined as the module density. Figure 8 shows the comparison between the density of each module obtained by the KISL algorithm and the base method, where a larger average module density is obtained on seven of the datasets and a larger number of modules with greater density are possessed. In addition, the top 3 modules with the highest module density on all eight datasets are found by our algorithm.

### 3.5 Comparison of module gene enrichment analysis

Co-expressed genes often act synergistically and participate in the same biological processes (van Dam et al., 2012). Therefore, algorithms that identify modules that are highly enriched for specific gene classes are more reasonable (Rau et al., 2013). To compare the average enrichment scores and stability of the algorithms, we use the recommended parameters of the WGCNA package for module identification, and to keep the number of modules identified by the two algorithms equal, the number of modules obtained by the WGCNA method is used to initialize the K values of our algorithms.

In the current analysis, we obtained the enrichment scores of each cluster in the functional annotation clustering of DAVID. The higher the enrichment score, the lower the *p*-value and therefore the more significant the enrichment. The module enrichment score is an important indicator to evaluate the rationality of a module. We discuss the average enrichment scores of modules from gene co-expression networks constructed by two different algorithms to measure the degree of enrichment of co-expression networks. As shown in Table 1, the modules from KISL have higher DAVID average enrichment scores in the six data sets, indicating that the division of their modules is more reasonable. Higher DAVID enrichment scores for each module can be viewed in (Supplementary Table S5), where the modules identified by KISL have the highest top 3 enrichment scores in the five datasets, and the top 3 modules have one or two enrichment scores in the other three datasets.

**TABLE 1:** Average DAVID enrichment score for each dataset.

	BLCA	BRCA	COAD	KIRC	LUAD	LUSC	STAD	PAAD
WGCNA	5.20	**9.53**	3.88	4.29	6.25	2.82	3.64	**5.98**
KISL	**6.11**	8.44	**4.12**	**5.64**	**7.36**	**6.55**	**4.63**	5.58

The bold words in Table 1 indicate the maximum value of the column, and the KISL algorithm obtains the maximum value on most data sets.

To verify whether the identification modules obtained by KISL are biologically meaningful, the highly enriched (Top 5) biological process (BP) terms of the network modules in GO terms were summarized for the LUSC sample, as shown in Table 2. Overall, the enrichment of GO terms shows the biological significance of the modules obtained by KISL.

**TABLE 2:** GOTERM BP on LUSC dataset.

Module	GOTERM BP
module0	O-glycan processing; innate immune response in mucosa; antibacterial humoral response; antimicrobial humoral immune response mediated by antimicrobial peptide; protein O-linked glycosylation
module1	DNA replication; DNA unwinding involved in DNA replication; spliceosomal snRNP assembly; mitochondrial translation; DNA-dependent DNA replication
module2	epithelial cell differentiation; epidermis development; intermediate filament organization; immunoglobulin production; keratinization
module3	cilium movement; flagellated sperm motility; microtubule-based movement; cilium assembly; outer dynein arm assembly
module4	cell division; chromosome segregation; mitotic spindle assembly checkpoint; mitotic cell cycle; mitotic spindle organization
module5	immunoglobulin production; immune response; positive regulation of B-cell activation; phagocytosis, recognition; phagocytosis, engulfment
module6	signal transduction; vasculogenesis; angiogenesis; positive regulation of angiogenesis; cell adhesion

## 4 Conclusion

Co-expression analysis is useful for exploring patterns of gene networks, identifying gene functional modules, and mining cancer-associated markers at the system level. By using the enriched information of the current sample as a constraint, we aim to perform semi-supervised clustering. Other clustering methods only take into account the algorithm parameters, not the sample itself. Therefore, we propose the KISL method to try to improve these methods. KISL algorithm measures linear and non-linear dependencies between genes by using distance correlation, which is appropriate for the complexity of the relationship between genes. In cases where outliers significantly influence the correlation coefficient value, distance correlation is a better alternative because it is distribution-free and better suited to complex relationships. Moreover, using biological knowledge based on GO terms to construct clustering constraints, a semi-supervised method is used to identify network modules, which can more effectively partition the network.

After comparing the silhouette coefficient, the Calinski-Harabasz index and the Davies-Bouldin index evaluation metric values of the modules identified by KISL with the widely used WGCNA, our algorithm obtained the best performance on eight real-world cancer sample datasets. The clustering produced by the method in this paper has a better clustering evaluation value, and the obtained gene modules have better aggregation. Based on enrichment analysis, the identified modules were effective in discovering modular structures in biological co-expression networks. The KISL method is a general method for analyzing biological co-expression networks based on similarity metrics.

In addition, we plan to incorporate more useful biological knowledge in the future, such as protein‒protein interaction networks and gene regulatory networks, which could allow us to better identify co-expressed gene modules. Genomics and transcriptomics are increasingly being applied to aid in clinical diagnosis and prognosis; thus, in addition to discussing module identification in co-expression network analysis, it is also important to develop effective methods for comparative network analysis. As part of our future research, we plan to explore how co-expression networks can be compared. It is our future goal to examine comparative methods of co-expression networks.

## Data Availability

The datasets presented in this study can be found in online repositories. The names of the repository/repositories and accession number(s) can be found in the article/Supplementary Material.

## References

[B1] Albert R. (2005). Scale-free networks in cell biology. J. Cell Sci. 118, 4947–4957. 10.1242/jcs.02714 16254242

[B2] Andrecut m. kauffman s. A. madni a. M. (2008). Evidence of scale-free topology in gene regulatory network of human tissues. Int. J. Mod. Phys. C 19, 283–290. 10.1142/s0129183108012091

[B3] Atiia A. A. Hopper C. Inoue K. Vidal S. Waldispühl J. (2020). Computational intractability law molds the topology of biological networks. Appl. Netw. Sci. 5, 34–22. 10.1007/s41109-020-00268-0

[B4] Bader G. D. Hogue C. W. V. (2003). An automated method for finding molecular complexes in large protein interaction networks. BMC Bioinforma. 4, 2. 10.1186/1471-2105-4-2 PMC149346 12525261

[B5] Barabási A.-L. Gulbahce N. Loscalzo J. (2011). Network medicine: A network-based approach to human disease. Nat. Rev. Genet. 12, 56–68. 10.1038/nrg2918 21164525 PMC3140052

[B6] Basu S. Banerjee A. Mooney R. J. (2004). “Active semi-supervision for pairwise constrained clustering,” in *Proceedings of the 2004 SIAM International Conference on data mining* 333–344 (Society for Industrial and Applied Mathematics). 10.1137/1.9781611972740.31

[B7] Caliński T. Harabasz J. (1974). A dendrite method for cluster analysis. Commun. Stat. 3 (1), 1–27. 10.1080/03610927408827101

[B8] Castro Sotos A. E. Vanhoof S. Van Den Noortgate W. Onghena P. (2009). The transitivity misconception of PEARSON’S correlation coefficient. Stat. Educ. Res. J. 8, 33–55. 10.52041/serj.v8i2.394

[B9] Davies D. L. Bouldin D. W. (1979). A cluster separation measure. *IEEE Trans. Pattern Anal. Mach. Intell.* PAMI- 1, 224–227. 10.1109/tpami.1979.4766909 21868852

[B10] Ferrando P. J. (2021). Seven decades of factor analysis: From yela to the present day. Psicothema 33, 378–385. 10.7334/psicothema2021.24 34297667

[B11] Gentleman R. C. Carey V. J. Bates D. M. Bolstad B. Dettling M. Dudoit S. (2004). Bioconductor: Open software development for computational biology and bioinformatics. Genome Biol. 5, R80. 10.1186/gb-2004-5-10-r80 15461798 PMC545600

[B12] GTEx Consortium (2015). Human genomics. The genotype-tissue expression (GTEx) pilot analysis: Multitissue gene regulation in humans. Science 348, 648–660. 10.1126/science.1262110 25954001 PMC4547484

[B13] GTEx Consortium (2013). The genotype-tissue expression (GTEx) project. Nat. Genet. 45, 580–585. 10.1038/ng.2653 23715323 PMC4010069

[B14] Hou J. Ye X. Feng W. Zhang Q. Han Y. Liu Y. (2022). Distance correlation application to gene co-expression network analysis. BMC Bioinforma. 23, 81. 10.1186/s12859-022-04609-x PMC8862277 35193539

[B15] Hou J. Ye X. Li C. Wang Y. K. (2021). K-module algorithm: An additional step to improve the clustering results of WGCNA Co-expression networks. Genes 12, 87. 10.3390/genes12010087 33445666 PMC7828115

[B16] Huang D. W. Sherman B. T. Lempicki R. A. (2009). Systematic and integrative analysis of large gene lists using DAVID bioinformatics resources. Nat. Protoc. 4, 44–57. 10.1038/nprot.2008.211 19131956

[B17] Hwang W. Cho Y.-R. Zhang A. Ramanathan M. (2006). A novel functional module detection algorithm for protein-protein interaction networks. Algorithms Mol. Biol. Amb. 1, 24. 10.1186/1748-7188-1-24 17147822 PMC1764415

[B18] Jia Z. Zhang X. (2022). Accurate determination of causalities in gene regulatory networks by dissecting downstream target genes. Front. Genet. 13, 923339. 10.3389/fgene.2022.923339 36568360 PMC9768335

[B19] Jiang X. Zhang X. (2022). Rsnet: Inferring gene regulatory networks by a redundancy silencing and network enhancement technique. BMC Bioinforma. 23, 165. 10.1186/s12859-022-04696-w PMC9074326 35524190

[B20] Langfelder P. Horvath S. (2008). Wgcna: an R package for weighted correlation network analysis. BMC Bioinforma. 9, 559. 10.1186/1471-2105-9-559 PMC2631488 19114008

[B21] Langfelder P. Zhang B. Horvath S. (2008). Defining clusters from a hierarchical cluster tree: The dynamic tree cut package for R. Bioinformatics 24, 719–720. 10.1093/bioinformatics/btm563 18024473

[B22] Love M. I. Huber W. Anders S. (2014). Moderated estimation of fold change and dispersion for RNA-seq data with DESeq2. Genome Biol. 15, 550. 10.1186/s13059-014-0550-8 25516281 PMC4302049

[B23] Nafis S. Kalaiarasan P. Brojen Singh R. K. Husain M. Bamezai R. N. K. (2015). Apoptosis regulatory protein-protein interaction demonstrates hierarchical scale-free fractal network. Brief. Bioinform. 16, 675–699. 10.1093/bib/bbu036 25256288

[B24] Pearson K. Galton F. V. I. I. (1895). Note on regression and inheritance in the case of two parents. Proc. R. Soc. Lond. 58, 240–242.

[B25] Pedregosa F. Varoquaux G. Gramfort A. Michel V. Thirion B. Weiss R. (2011). Scikit-learn: Machine learning in Python. J. Mach. Learn. Res. 12, 2825–2830.

[B26] Ramos-Carreño C. Torrecilla J. L. (2022). dcor: distance correlation and energy statistics in Python. Orig. Softw. Publ. 22, 101326. 10.5281/zenodo.7484447

[B27] Rau C. D. Wisniewski N. Orozco L. D. Bennett B. Weiss J. Lusis A. J. (2013). Maximal information component analysis: A novel non-linear network analysis method. Front. Genet. 4, 28. 10.3389/fgene.2013.00028 23487572 PMC3594742

[B28] Ravasz E. Somera A. L. Mongru D. A. Oltvai Z. N. Barabási A. L. (2002). Hierarchical organization of modularity in metabolic networks. Science 297, 1551–1555. 10.1126/science.1073374 12202830

[B29] RousseeuwSilhouettes P. J. (1987). Silhouettes: A graphical aid to the interpretation and validation of cluster analysis. J. Comput. Appl. Math. 20, 53–65. 10.1016/0377-0427(87)90125-7

[B30] Ruan J. Zhang W. (2008). Identifying network communities with a high resolution. Phys. Rev. E Stat. Nonlin. Soft Matter Phys. 77, 016104. 10.1103/PhysRevE.77.016104 18351912

[B31] Swisher L. L. Beckstead J. W. Bebeau M. J. (2004). Factor analysis as a tool for survey analysis using a professional role orientation inventory as an example. Phys. Ther. 84, 784–799. 10.1093/ptj/84.9.784 15330692

[B32] Székely G. J. Rizzo M. L. Bakirov N. K. (2007). Measuring and testing dependence by correlation of distances. Ann. Stat. 35, 2769–2794. 10.1214/009053607000000505

[B33] Székely G. J. Rizzo M. L. (2009). Brownian distance covariance. Ann. Appl. Stat. 3, 1236–1265. 10.1214/09-aoas312 PMC2889501 20574547

[B34] van Dam S. Cordeiro R. Craig T. van Dam J. Wood S. H. de Magalhães J. P. (2012). GeneFriends: An online co-expression analysis tool to identify novel gene targets for aging and complex diseases. BMC Genomics 13, 535. 10.1186/1471-2164-13-535 23039964 PMC3495651

[B35] Virtanen P. Gommers R. Oliphant T. E. Haberland M. Reddy T. Cournapeau D. (2020). SciPy 1.0: Fundamental algorithms for scientific computing in Python. Nat. Methods 17, 261–272. 10.1038/s41592-019-0686-2 32015543 PMC7056644

[B36] Yip A. M. Horvath S. (2007). Gene network interconnectedness and the generalized topological overlap measure. BMC Bioinforma. 8, 22. 10.1186/1471-2105-8-22 PMC1797055 17250769

[B37] Yip A. M. Horvath S. (2007). Gene network interconnectedness and the generalized topological overlap measure. BMC Bioinforma. 8, 22. 10.1186/1471-2105-8-22 PMC1797055 17250769

[B38] Yu G. Wang L.-G. Han Y. He Q.-Y. (2012). clusterProfiler: an R package for comparing biological themes among gene clusters. Omics J. Integr. Biol. 16, 284–287. 10.1089/omi.2011.0118 PMC3339379 22455463

[B39] Zhang B. Horvath S. (2005). A general framework for weighted gene co-expression network analysis. Stat. Appl. Genet. Mol. Biol. 4, 17. 10.2202/1544-6115.1128 16646834

