# Supplementary material for: KISL: knowledge-injected semi-supervised learning for biological co-expression network modules
Source: Front Genet. 2023 May 2;14:1151962. doi: 10.3389/fgene.2023.1151962 (PMC10185879; doi:10.3389/fgene.2023.1151962)
Supplement: Supplementary file 1 [file Table5.pdf]

Tabls S5: The gene number and DAVID enrichment score in each module

BLCA cancer and Pearson-based WGCNA

| | module0 | module1 | module2 | module3 | module4 | module5 | module6 | module7 | module8 | module9 | module10 |
|---|---|---|---|---|---|---|---|---|---|---|---|
| number of gene | 846 | 1603 | 920 | 737 | 454 | 242 | 186 | 180 | 172 | 152 | 122 |
| Cluster 0 | 21.45 | 8.19 | 46.27 | 5.21 | 6.13 | 10.27 | 31.47 | 3.62 | 0.86 | 6.78 | 1.25 |
| Cluster 1 | 5.13 | 5.31 | 21.84 | 2.32 | 2.71 | 5.89 | 27.35 | 2.84 | 0.81 | 2.73 | 0.35 |
| Cluster 2 | 4.68 | 4.94 | 17.51 | 1.51 | 2.65 | 5.72 | 11.92 | 2.65 | 0.72 | 2.71 | 0.30 |
| Cluster 3 | 4.12 | 4.55 | 15.23 | 1.49 | 2.15 | 5.12 | 9.43 | 2.28 | 0.42 | 2.53 | 0.22 |
| Cluster 4 | 3.74 | 4.30 | 8.87 | 1.01 | 2.13 | 4.39 | 8.05 | 1.86 | 0.22 | 1.76 | 0.19 |
| Cluster 5 | 2.47 | 3.94 | 8.60 | 0.98 | 1.96 | 4.14 | 4.74 | 1.45 | * | 1.18 | * |
| Cluster 6 | 2.32 | 3.09 | 7.65 | 0.89 | 1.86 | 4.02 | 4.65 | 1.27 | * | 1.14 | * |
| Cluster 7 | 2.15 | 3.03 | 7.65 | 0.79 | 1.34 | 3.78 | 4.24 | 1.20 | * | 1.00 | * |
| Cluster 8 | 1.99 | 2.78 | 6.82 | 0.77 | 1.30 | 3.53 | 3.84 | 1.10 | * | 0.96 | * |
| Cluster 9 | 1.94 | 2.73 | 5.78 | 0.69 | 1.24 | 3.53 | 2.69 | 1.01 | * | 0.94 | * |
| mean score | 5.00 | 4.29 | **14.62** | 1.57 | 2.35 | 5.04 | **10.84** | 1.93 | 0.61 | 2.17 | 0.46 |

| | module11 | module12 | module13 | module14 | module15 | module16 | module17 |
|---|---|---|---|---|---|---|---|
| number of gene | 120 | 115 | 92 | 86 | 84 | 76 | 66 |
| Cluster 0 | 21.13 | 16.45 | * | 71.76 | 3.70 | 4.98 | 1.31 |
| Cluster 1 | 7.23 | 15.55 | * | 58.61 | 3.42 | 4.13 | 1.16 |
| Cluster 2 | 4.71 | 4.53 | * | 39.87 | 1.14 | 3.54 | 0.89 |
| Cluster 3 | 4.38 | 3.83 | * | 21.27 | 1.02 | 2.79 | 0.87 |
| Cluster 4 | 3.84 | 3.63 | * | 7.24 | 0.96 | 1.93 | 0.76 |
| Cluster 5 | 3.77 | 2.55 | * | 3.29 | 0.71 | 1.93 | 0.63 |
| Cluster 6 | 3.48 | 2.50 | * | 0.55 | 0.65 | 1.84 | 0.36 |
| Cluster 7 | 2.75 | 2.01 | * | * | 0.31 | 1.56 | 0.11 |
| Cluster 8 | 2.72 | 1.96 | * | * | * | 1.44 | 0.02 |
| Cluster 9 | 2.53 | 1.74 | * | * | * | 0.37 | * |
| mean score | 5.65 | 5.48 | * | **28.94** | 1.49 | 2.45 | 0.68 |

BLCA cancer and KISL

| | module0 | module1 | module2 | module3 | module4 | module5 | module6 | module7 | module8 | module9 | module10 |
|---|---|---|---|---|---|---|---|---|---|---|---|
| number of gene | 242 | 385 | 470 | 1202 | 321 | 613 | 515 | 383 | 203 | 519 | 86 |
| Cluster 0 | 6.34 | 7.42 | 1.85 | 13.02 | 7.18 | 2.08 | 3.14 | 6.49 | 9.43 | 4.67 | 83.53 |
| Cluster 1 | 1.85 | 4.37 | 1.74 | 7.09 | 6.68 | 1.89 | 1.86 | 4.31 | 8.25 | 2.99 | 67.25 |
| Cluster 2 | 0.98 | 4.37 | 1.65 | 5.40 | 6.63 | 1.70 | 1.41 | 3.52 | 4.23 | 1.98 | 53.17 |
| Cluster 3 | 0.67 | 3.98 | 1.32 | 4.06 | 5.46 | 1.60 | 0.84 | 3.22 | 3.89 | 1.94 | 48.68 |
| Cluster 4 | 0.57 | 3.59 | 1.26 | 3.79 | 5.17 | 1.59 | 0.72 | 2.93 | 3.78 | 1.86 | 23.91 |
| Cluster 5 | 0.35 | 3.54 | 1.17 | 3.37 | 4.29 | 1.48 | 0.35 | 2.35 | 3.72 | 1.79 | 6.08 |
| Cluster 6 | 0.33 | 3.50 | 1.16 | 3.34 | 3.89 | 1.19 | 0.25 | 2.21 | 2.24 | 1.34 | 3.29 |
| Cluster 7 | 0.10 | 3.31 | 1.02 | 2.69 | 3.85 | 1.13 | 0.20 | 1.87 | 2.02 | 1.31 | * |
| Cluster 8 | 0.01 | 2.53 | 1.00 | 2.58 | 3.63 | 0.95 | 0.16 | 1.82 | 1.92 | 1.27 | * |
| Cluster 9 | * | 2.50 | 0.99 | 2.42 | 3.15 | 0.95 | 0.13 | 1.75 | 1.89 | 1.24 | * |
| mean score | 1.24 | 3.91 | 1.32 | 4.78 | 4.99 | 1.46 | 0.91 | 3.05 | 4.14 | 2.04 | **40.84** |

| | module11 | module12 | module13 | module14 | module15 | module16 | module17 |
|---|---|---|---|---|---|---|---|
| number of gene | 498 | 91 | 109 | 197 | 135 | 177 | 107 |
| Cluster 0 | 2.35 | 14.66 | 6.4 | 13.42 | 26.88 | 60.8 | 1.81 |
| Cluster 1 | 1.59 | 3.82 | 5.44 | 11.47 | 21.03 | 28.99 | 1.13 |
| Cluster 2 | 1.42 | 3.75 | 3.17 | 3.37 | 9.44 | 15.84 | 0.98 |
| Cluster 3 | 1.14 | 3.72 | 2.91 | 2.85 | 8.97 | 15.08 | 0.88 |
| Cluster 4 | 1.13 | 3.13 | 2.84 | 2.66 | 7.75 | 13.71 | 0.80 |
| Cluster 5 | 0.95 | 3.08 | 2.82 | 2.63 | 6.51 | 13.17 | 0.75 |
| Cluster 6 | 0.91 | 2.87 | 1.38 | 2.02 | 5.92 | 13.13 | 0.57 |
| Cluster 7 | 0.88 | 2.76 | 1.36 | 1.95 | 5.57 | 5.89 | 0.31 |
| Cluster 8 | 0.86 | 2.60 | 1.21 | 1.93 | 5.25 | 4.68 | 0.29 |
| Cluster 9 | 0.85 | 1.71 | 0.89 | 1.92 | 4.49 | 4.66 | 0.29 |
| mean score | 1.21 | 4.21 | 2.84 | 4.42 | **10.18** | **17.60** | 0.78 |

BRCA cancer and Pearson-based WGCNA

| | module0 | module1 | module2 | module3 | module4 | module5 | module6 | module7 | module8 | module9 |
|---|---|---|---|---|---|---|---|---|---|---|
| number of gene | 632 | 2704 | 2055 | 511 | 479 | 381 | 123 | 123 | 66 | 64 |
| Cluster 0 | 15.8 | 16.89 | 6.14 | 51.99 | 142.79 | 4.08 | 3.76 | 20.63 | 7.86 | 4.01 |
| Cluster 1 | 5.88 | 12.85 | 4.93 | 24.88 | 73.75 | 3.60 | * | 7.11 | 4.83 | 3.42 |
| Cluster 2 | 5.23 | 5.08 | 4.01 | 22.39 | 69.39 | 2.95 | * | 5.52 | 4.23 | 3.10 |
| Cluster 3 | 2.73 | 5.01 | 3.52 | 11.55 | 64.22 | 2.72 | * | 3.72 | 1.02 | 2.60 |
| Cluster 4 | 2.70 | 4.57 | 3.11 | 10.83 | 46.36 | 2.60 | * | 2.66 | 0.97 | 2.35 |
| Cluster 5 | 2.60 | 3.91 | 2.44 | 8.81 | 32.17 | 2.55 | * | 2.66 | 0.62 | 1.59 |
| Cluster 6 | 2.37 | 3.85 | 2.43 | 8.69 | 30.11 | 2.33 | * | 2.56 | 0.28 | 1.39 |
| Cluster 7 | 2.21 | 3.79 | 2.23 | 7.51 | 17.4 | 2.29 | * | 2.52 | 0.05 | 1.33 |
| Cluster 8 | 2.16 | 3.43 | 1.83 | 6.87 | 9.20 | 2.15 | * | 2.24 | * | 1.07 |
| Cluster 9 | 1.96 | 3.39 | 1.81 | 5.79 | 7.19 | 1.96 | * | 1.64 | * | 0.86 |
| mean score | 4.36 | **6.28** | 3.24 | **15.93** | **49.26** | 2.72 | 3.76 | 5.13 | 2.48 | 2.17 |

BRCA cancer and KISL

| | module0 | module1 | module2 | module3 | module4 | module5 | module6 | module7 | module8 | module9 |
|---|---|---|---|---|---|---|---|---|---|---|
| number of gene | 155 | 1203 | 1104 | 1229 | 406 | 990 | 1127 | 369 | 232 | 323 |
| Cluster 0 | 2.39 | 8.47 | 23.41 | 5.67 | 1.36 | 7.73 | 7.37 | 149.72 | 64.19 | 2.51 |
| Cluster 1 | 1.73 | 4.80 | 19.91 | 4.22 | 1.36 | 6.30 | 5.75 | 80.01 | 30.78 | 2.15 |
| Cluster 2 | 1.64 | 3.00 | 7.96 | 3.42 | 1.23 | 3.31 | 4.67 | 62.03 | 15.29 | 2.05 |
| Cluster 3 | 1.03 | 2.85 | 5.09 | 2.71 | 1.22 | 3.20 | 2.79 | 61.29 | 13.16 | 1.87 |
| Cluster 4 | 0.70 | 2.64 | 4.59 | 2.22 | 1.17 | 3.19 | 2.71 | 31.73 | 12.42 | 1.86 |
| Cluster 5 | 0.55 | 2.50 | 4.27 | 2.21 | 1.09 | 2.95 | 2.43 | 18.70 | 11.51 | 1.41 |
| Cluster 6 | 0.47 | 2.22 | 4.12 | 1.78 | 0.93 | 2.59 | 2.39 | 9.13 | 8.44 | 1.28 |
| Cluster 7 | 0.42 | 2.17 | 3.84 | 1.63 | 0.89 | 2.55 | 2.27 | 4.62 | 5.85 | 1.26 |
| Cluster 8 | 0.20 | 2.06 | 3.49 | 1.62 | 0.84 | 2.28 | 2.22 | 3.90 | 5.73 | 0.92 |
| Cluster 9 | 0.15 | 2.04 | 2.76 | 1.57 | 0.70 | 2.21 | 2.13 | 3.44 | 5.39 | 0.86 |
| mean score | 0.93 | 3.28 | **7.94** | 2.70 | 1.08 | 3.63 | 3.47 | **42.46** | **17.28** | 1.62 |

COAD cancer and Pearson-based WGCNA

| | module0 | module1 | module2 | module3 | module4 | module5 | module6 | module7 | module8 | module9 | module10 | module11 | module12 |
|---|---|---|---|---|---|---|---|---|---|---|---|---|---|
| number of gene | 2284 | 1782 | 852 | 396 | 268 | 225 | 225 | 170 | 135 | 107 | 103 | 92 | 84 |
| Cluster 0 | 55.57 | 7.67 | 45.08 | 10.2 | 1.09 | 8.23 | 2.45 | 4.56 | 0.89 | 4.08 | 1.99 | 8.41 | 4.94 |
| Cluster 1 | 11.62 | 6.97 | 22.39 | 8.79 | 0.8 | 5.21 | 0.99 | 2.04 | * | 3.49 | 1.78 | 2.03 | 4.2 |
| Cluster 2 | 8.75 | 6.32 | 17.04 | 4.32 | 0.59 | 2.98 | 0.94 | 1.17 | * | 2.6 | 1.76 | 1.81 | 3.36 |
| Cluster 3 | 7.68 | 5.44 | 14.92 | 3.74 | 0.58 | 2.68 | 0.77 | 1.1 | * | 2.01 | 1.74 | 1.74 | 2.85 |
| Cluster 4 | 7.45 | 5.38 | 11.93 | 3.3 | 0.37 | 2.66 | 0.77 | 0.98 | * | 0.94 | 1.5 | 1.72 | 2.57 |
| Cluster 5 | 7.10 | 4.99 | 9.13 | 3.13 | 0.26 | 2.43 | 0.67 | 0.81 | * | 0.66 | 1.09 | 1.33 | 2.46 |
| Cluster 6 | 5.54 | 4.48 | 8.33 | 2.54 | 0.12 | 2.20 | 0.5 | 0.54 | * | 0.52 | 0.85 | 1.23 | 2.35 |
| Cluster 7 | 4.76 | 4.42 | 6.56 | 2.43 | 0.1 | 2.07 | 0.35 | 0.47 | * | 0.49 | 0.80 | 1.22 | 1.98 |
| Cluster 8 | 4.23 | 4.23 | 6.46 | 2.34 | 0.07 | 2.05 | 0.32 | 0.18 | * | 0.45 | 0.80 | 0.71 | 1.87 |
| Cluster 9 | 3.87 | 4.04 | 6.37 | 2.33 | 0.01 | 2.05 | 0.19 | 0.15 | * | 0.17 | 0.76 | 0.6 | 1.71 |
| mean score | **11.66** | **5.39** | **14.82** | 4.31 | 0.40 | 3.26 | 0.80 | 1.20 | 0.89 | 1.54 | 1.31 | 2.08 | 2.83 |

COAD cancer and KISL

| | module0 | module1 | module2 | module3 | module4 | module5 | module6 | module7 | module8 | module9 | module10 | module11 | module12 |
|---|---|---|---|---|---|---|---|---|---|---|---|---|---|
| number of gene | 614 | 557 | 632 | 80 | 539 | 369 | 18 | 1447 | 195 | 936 | 82 | 374 | 880 |
| Cluster 0 | 49.91 | 4.87 | 4.70 | * | 4.73 | 1.81 | 9.61 | 8.86 | 5.28 | 46.04 | 2.12 | 6.23 | 9.23 |
| Cluster 1 | 30.48 | 4.62 | 3.41 | * | 4.21 | 1.69 | 5.66 | 7.15 | 1.54 | 9.17 | 1.84 | 6.08 | 7.55 |
| Cluster 2 | 22.15 | 4.43 | 2.36 | * | 2.95 | 1.45 | 4.80 | 4.89 | 1.19 | 8.27 | 1.22 | 3.25 | 6.03 |
| Cluster 3 | 14.36 | 3.00 | 2.02 | * | 2.60 | 1.43 | 2.41 | 3.46 | 0.95 | 8.19 | 0.93 | 2.83 | 5.98 |
| Cluster 4 | 10.76 | 2.06 | 2.00 | * | 1.90 | 1.42 | 1.10 | 3.39 | 0.85 | 6.92 | 0.90 | 2.03 | 4.62 |
| Cluster 5 | 9.45 | 2.01 | 1.93 | * | 1.86 | 1.33 | 0.12 | 3.07 | 0.59 | 5.51 | 0.77 | 2.03 | 3.87 |
| Cluster 6 | 7.34 | 1.85 | 1.90 | * | 1.77 | 1.29 | * | 2.96 | 0.52 | 5.14 | 0.76 | 1.99 | 3.78 |
| Cluster 7 | 6.58 | 1.83 | 1.88 | * | 1.52 | 1.19 | * | 2.35 | 0.51 | 4.40 | 0.74 | 1.94 | 3.59 |
| Cluster 8 | 5.20 | 1.82 | 1.85 | * | 1.34 | 1.18 | * | 1.99 | 0.34 | 4.29 | 0.53 | 1.70 | 3.55 |
| Cluster 9 | 5.09 | 1.81 | 1.79 | * | 1.16 | 1.14 | * | 1.87 | 0.24 | 3.70 | 0.50 | 1.67 | 3.54 |
| mean score | **16.13** | 2.83 | 2.38 | * | 2.40 | 1.39 | 3.95 | 4.00 | 1.20 | **10.16** | 1.03 | 2.98 | **5.17** |

KIRC cancer and Pearson-based WGCNA

| | module0 | module1 | module2 | module3 | module4 | module5 | module6 | module7 | module8 | module9 | module10 |
|---|---|---|---|---|---|---|---|---|---|---|---|
| number of gene | 46 | 1540 | 485 | 358 | 350 | 304 | 276 | 259 | 254 | 242 | 235 |
| Cluster 0 | 4.08 | 9.64 | 16.56 | 13.51 | 23.32 | 1.38 | 5.18 | 13.99 | 9.89 | 61.82 | 1.57 |
| Cluster 1 | 2.92 | 3.00 | 14.43 | 9.42 | 17.76 | 1.28 | 2.23 | 5.77 | 2.38 | 5.96 | 1.55 |
| Cluster 2 | 1.47 | 2.48 | 7.58 | 6.77 | 5.99 | 1.25 | 1.97 | 3.69 | 2.25 | 5.76 | 1.41 |
| Cluster 3 | 1.26 | 2.25 | 7.20 | 6.67 | 5.83 | 1.12 | 1.68 | 3.02 | 2.21 | 4.41 | 1.4 |
| Cluster 4 | 0.99 | 2.18 | 4.00 | 3.60 | 4.89 | 1.11 | 1.56 | 1.95 | 2.03 | 4.23 | 1.34 |
| Cluster 5 | 0.77 | 2.00 | 3.11 | 3.48 | 4.46 | 1.07 | 1.54 | 1.85 | 1.74 | 4.10 | 1.10 |
| Cluster 6 | 0.64 | 1.98 | 2.93 | 3.27 | 4.32 | 0.95 | 1.49 | 1.28 | 1.65 | 3.91 | 1.01 |
| Cluster 7 | * | 1.87 | 2.89 | 3.27 | 3.57 | 0.91 | 1.37 | 1.26 | 1.61 | 2.81 | 0.74 |
| Cluster 8 | * | 1.78 | 2.44 | 2.89 | 2.66 | 0.88 | 1.23 | 1.24 | 1.57 | 2.67 | 0.73 |
| Cluster 9 | * | 1.77 | 2.36 | 2.84 | 2.60 | 0.86 | 1.20 | 1.23 | 1.51 | 2.14 | 0.71 |
| mean score | 1.52 | 2.90 | 6.35 | 5.57 | **7.54** | 1.08 | 1.95 | 3.53 | 2.68 | **9.78** | 1.16 |

| | module11 | module12 | module13 | module14 | module15 | module16 | module17 | module18 | module19 |
|---|---|---|---|---|---|---|---|---|---|
| number of gene | 196 | 134 | 132 | 100 | 100 | 81 | 67 | 67 | 65 |
| Cluster 0 | 2.08 | 93.23 | 2.53 | 13.76 | 2.41 | 2.78 | 5.22 | 3.80 | * |
| Cluster 1 | 1.81 | 73.88 | 2.35 | 5.65 | 1.49 | 1.66 | 3.93 | 2.02 | * |
| Cluster 2 | 1.25 | 48.27 | 1.81 | 3.89 | 1.46 | 1.44 | 3.61 | 2.01 | * |
| Cluster 3 | 1.21 | 29.93 | 1.30 | 2.98 | 0.47 | 1.42 | 3.56 | 1.74 | * |
| Cluster 4 | 1.03 | 6.78 | 0.90 | 2.89 | 0.26 | 1.28 | 3.46 | 0.97 | * |
| Cluster 5 | 1.00 | 3.76 | 0.72 | 2.48 | 0.14 | 1.00 | 3.38 | 0.54 | * |
| Cluster 6 | 0.79 | 2.48 | 0.64 | 2.38 | * | 0.98 | 2.94 | 0.40 | * |
| Cluster 7 | 0.75 | 0.92 | 0.57 | 2.33 | * | 0.88 | 2.84 | 0.13 | * |
| Cluster 8 | 0.68 | 0.03 | 0.55 | 2.10 | * | 0.85 | 0.69 | 0.09 | * |
| Cluster 9 | 0.67 | * | 0.42 | 1.95 | * | 0.74 | 0.11 | * | * |
| mean score | 1.13 | **28.81** | 1.18 | 4.04 | 1.04 | 1.30 | 2.97 | 1.30 | * |

KIRC cancer and KISL

| | module0 | module1 | module2 | module3 | module4 | module5 | module6 | module7 | module8 | module9 | module10 |
|---|---|---|---|---|---|---|---|---|---|---|---|
| number of gene | 273 | 647 | 131 | 510 | 107 | 346 | 362 | 184 | 459 | 219 | 909 |
| Cluster 0 | 56.49 | 15.49 | 1.98 | 11.23 | 1.02 | 1.47 | 1.95 | 0.60 | 2.60 | 1.26 | 18.58 |
| Cluster 1 | 39.42 | 15.23 | 1.56 | 4.61 | 0.84 | 1.25 | 1.40 | 0.48 | 2.01 | 0.71 | 5.62 |
| Cluster 2 | 6.76 | 5.40 | 1.55 | 2.88 | 0.59 | 1.00 | 1.29 | 0.46 | 1.95 | 0.53 | 2.91 |
| Cluster 3 | 4.39 | 4.17 | 1.47 | 2.51 | 0.18 | 0.53 | 1.14 | 0.36 | 1.78 | 0.48 | 2.86 |
| Cluster 4 | 4.21 | 2.69 | 1.27 | 2.01 | 0.09 | 0.46 | 1.06 | 0.32 | 1.72 | 0.38 | 2.71 |
| Cluster 5 | 3.94 | 2.53 | 1.26 | 1.88 | * | 0.31 | 0.96 | 0.31 | 1.65 | 0.37 | 2.56 |
| Cluster 6 | 3.03 | 2.31 | 1.10 | 1.82 | * | 0.14 | 0.76 | 0.3 | 1.56 | 0.34 | 2.28 |
| Cluster 7 | 2.17 | 2.11 | 1.07 | 1.78 | * | * | 0.73 | 0.28 | 1.43 | 0.28 | 2.16 |
| Cluster 8 | 1.98 | 1.92 | 0.94 | 1.71 | * | * | 0.72 | 0.17 | 1.37 | 0.24 | 2.14 |
| Cluster 9 | 1.63 | 1.85 | 0.84 | 1.60 | * | * | 0.72 | * | 1.30 | 0.13 | 2.13 |
| mean score | **12.40** | 5.37 | 1.30 | 3.20 | 0.45 | 0.74 | 1.07 | 0.36 | 1.74 | 0.47 | 4.40 |

| | module11 | module12 | module13 | module14 | module15 | module16 | module17 | module18 | module19 |
|---|---|---|---|---|---|---|---|---|---|
| number of gene | 260 | 129 | 107 | 35 | 183 | 67 | 74 | 108 | 181 |
| Cluster 0 | 12.96 | 2.94 | 102.32 | 3.57 | 1.98 | 1.54 | 25.86 | 3.81 | 19.85 |
| Cluster 1 | 12.85 | 1.95 | 70.45 | 2.91 | 1.63 | 1.48 | 19.43 | 3.43 | 19.19 |
| Cluster 2 | 5.67 | 1.08 | 52.22 | 2.87 | 1.52 | 1.41 | 8.70 | 2.89 | 7.78 |
| Cluster 3 | 4.53 | 0.75 | 31.01 | 2.48 | 1.50 | 1.36 | 4.29 | 2.86 | 7.27 |
| Cluster 4 | 4.38 | 0.72 | 7.09 | 2.27 | 1.40 | 1.18 | 2.61 | 2.76 | 7.07 |
| Cluster 5 | 3.22 | 0.43 | * | 2.16 | 1.29 | 1.01 | 2.00 | 2.35 | 6.72 |
| Cluster 6 | 3.17 | 0.31 | * | 0.99 | 1.11 | 0.92 | 1.45 | 2.10 | 5.60 |
| Cluster 7 | 2.62 | 0.18 | * | 0.84 | 0.95 | 0.87 | 1.22 | 1.94 | 4.53 |
| Cluster 8 | 2.52 | 0.18 | * | * | 0.50 | 0.33 | 0.67 | 1.57 | 4.45 |
| Cluster 9 | 2.38 | 0.10 | * | * | 0.46 | 0.12 | 0.44 | 1.54 | 3.40 |
| mean score | 5.43 | 0.86 | **52.62** | 2.26 | 1.23 | 1.02 | 6.67 | 2.52 | **8.59** |

LUAD cancer and Pearson-based WGCNA

| | module0 | module1 | module10 | module11 | module2 | module3 | module4 | module5 | module6 | module7 | module8 | module9 |
|---|---|---|---|---|---|---|---|---|---|---|---|---|
| number of gene | 1307 | 2265 | 67 | 59 | 1540 | 663 | 287 | 165 | 112 | 109 | 88 | 87 |
| Cluster 0 | 41.34 | 8.90 | 2.43 | * | 11.41 | 33.57 | 2.13 | 111.47 | 7.53 | * | 1.48 | 5.11 |
| Cluster 1 | 34.87 | 7.41 | 1.83 | * | 9.72 | 20.33 | 2.01 | 76.68 | 5.66 | * | 1.28 | 4.25 |
| Cluster 2 | 7.95 | 6.28 | 1.31 | * | 9.58 | 9.87 | 1.98 | 68.18 | 4.52 | * | 1.27 | 2.45 |
| Cluster 3 | 6.70 | 6.12 | 1.28 | * | 6.31 | 9.36 | 1.94 | 25.88 | 3.39 | * | 1.25 | 2.07 |
| Cluster 4 | 6.46 | 5.59 | 1.27 | * | 6.04 | 9.04 | 1.94 | 9.13 | 2.50 | * | 1.18 | 1.54 |
| Cluster 5 | 5.94 | 4.38 | 1.25 | * | 5.84 | 8.25 | 1.82 | 6.79 | 1.59 | * | 1.04 | 1.25 |
| Cluster 6 | 4.27 | 4.29 | 0.40 | * | 5.48 | 7.22 | 1.51 | 4.01 | 1.59 | * | 0.92 | 0.04 |
| Cluster 7 | 3.63 | 4.27 | 0.13 | * | 4.77 | 6.30 | 1.50 | 1.25 | 0.81 | * | 0.77 | * |
| Cluster 8 | 3.57 | 2.97 | 0.09 | * | 4.32 | 6.28 | 1.41 | 0.19 | 0.69 | * | 0.70 | * |
| Cluster 9 | 3.40 | 2.64 | * | * | 3.75 | 6.07 | 1.13 | 0.01 | 0.65 | * | 0.56 | * |
| mean score | **11.81** | 5.28 | 1.11 | * | 6.72 | **11.63** | 1.74 | **30.36** | 2.89 | * | 1.04 | 2.39 |

LUAD cancer and KISL

| | module0 | module1 | module2 | module3 | module4 | module5 | module6 | module7 | module8 | module9 | module10 | module11 |
|---|---|---|---|---|---|---|---|---|---|---|---|---|
| number of gene | 126 | 1149 | 675 | 683 | 42 | 824 | 653 | 1250 | 428 | 48 | 198 | 673 |
| Cluster 0 | 107.7 | 7.2 | 21.28 | 2.8 | 4.92 | 7.47 | 8.11 | 4.26 | 2.87 | 1.87 | 54.69 | 4.8 |
| Cluster 1 | 80.61 | 2.41 | 11.88 | 2.29 | 2.59 | 2.92 | 4.07 | 4.16 | 2.68 | 1.04 | 31.39 | 4.45 |
| Cluster 2 | 62.9 | 2.33 | 9.91 | 2.24 | 2.24 | 2.89 | 2.56 | 3.81 | 2.09 | 0.95 | 14.01 | 4.08 |
| Cluster 3 | 27.42 | 2.29 | 7.66 | 2.15 | 2.16 | 2.75 | 2.46 | 2.73 | 2.02 | 0.55 | 13.99 | 3.85 |
| Cluster 4 | 8.96 | 2.16 | 5.84 | 1.44 | 1.30 | 2.08 | 2.41 | 2.48 | 1.86 | 0.40 | 12.96 | 3.83 |
| Cluster 5 | 4.62 | 1.90 | 5.25 | 1.38 | 0.49 | 1.93 | 2.02 | 2.42 | 1.82 | 0.36 | 12.22 | 3.61 |
| Cluster 6 | 2.73 | 1.42 | 4.94 | 1.33 | * | 1.77 | 1.65 | 2.13 | 1.78 | 0.18 | 11.10 | 3.49 |
| Cluster 7 | * | 1.38 | 4.12 | 1.30 | * | 1.66 | 1.59 | 1.99 | 1.70 | 0.12 | 8.57 | 3.47 |
| Cluster 8 | * | 1.37 | 3.75 | 1.28 | * | 1.65 | 1.58 | 1.95 | 1.56 | * | 7.97 | 3.47 |
| Cluster 9 | * | 1.33 | 3.37 | 1.24 | * | 1.63 | 1.46 | 1.80 | 1.55 | * | 5.82 | 3.25 |
| mean score | **42.13** | 2.38 | **7.80** | 1.74 | 2.28 | 2.68 | 2.79 | 2.77 | 1.99 | 0.68 | **17.27** | 3.83 |

LUSC cancer and Pearson-based WGCNA

| | module0 | module1 | module2 | module3 | module4 | module5 | module6 |
|---|---|---|---|---|---|---|---|
| number of gene | 6379 | 789 | 749 | 350 | 216 | 155 | 110 |
| Cluster 0 | * | 4.62 | 7.78 | 1.75 | 24.07 | 2.94 | 1.71 |
| Cluster 1 | * | 3.81 | 7.47 | 1.33 | 22.75 | 1.62 | 1.46 |
| Cluster 2 | * | 2.14 | 7.16 | 1.16 | 14.23 | 1.45 | 1.30 |
| Cluster 3 | * | 1.80 | 6.19 | 1.07 | 8.23 | 1.40 | 1.17 |
| Cluster 4 | * | 1.79 | 4.66 | 0.91 | 5.81 | 1.36 | 0.96 |
| Cluster 5 | * | 1.77 | 4.29 | 0.80 | 5.35 | 1.08 | 0.90 |
| Cluster 6 | * | 1.59 | 3.44 | 0.56 | 4.09 | 0.97 | 0.81 |
| Cluster 7 | * | 1.37 | 3.41 | 0.45 | 3.22 | 0.96 | 0.79 |
| Cluster 8 | * | 1.30 | 3.23 | 0.45 | 2.55 | 0.89 | 0.72 |
| Cluster 9 | * | 1.29 | 3.18 | 0.42 | 2.12 | 0.82 | 0.71 |
| mean score | * | **2.15** | **5.08** | 0.89 | **9.24** | 1.35 | 1.05 |

LUSC cancer and KISL

| | module0 | module1 | module2 | module3 | module4 | module5 | module6 |
|---|---|---|---|---|---|---|---|
| number of gene | 2156 | 1556 | 2172 | 137 | 1018 | 808 | 901 |
| Cluster 0 | 7.27 | 9.68 | 22.67 | 18.94 | 19.47 | 29.92 | 6.49 |
| Cluster 1 | 5.22 | 4.29 | 12.05 | 16.02 | 8.09 | 25.69 | 6.18 |
| Cluster 2 | 3.08 | 3.87 | 11.73 | 8.44 | 5.60 | 19.97 | 5.84 |
| Cluster 3 | 2.69 | 3.50 | 11.66 | 6.70 | 5.24 | 13.06 | 4.78 |
| Cluster 4 | 2.31 | 3.14 | 11.15 | 3.04 | 4.96 | 6.26 | 4.66 |
| Cluster 5 | 2.31 | 2.74 | 8.27 | 2.83 | 4.26 | 3.94 | 4.50 |
| Cluster 6 | 2.30 | 2.72 | 7.88 | 2.18 | 4.10 | 3.67 | 4.15 |
| Cluster 7 | 2.13 | 2.60 | 7.62 | 2.06 | 3.96 | 2.53 | 4.11 |
| Cluster 8 | 1.92 | 2.54 | 7.40 | 1.71 | 3.70 | 2.21 | 3.76 |
| Cluster 9 | 1.73 | 2.46 | 6.77 | 1.61 | 2.95 | 2.11 | 3.41 |
| mean score | 3.10 | 3.75 | **10.72** | **6.35** | 6.23 | **10.94** | 4.79 |

PAAD cancer and Pearson-based WGCNA

| | module0 | module1 | module2 | module3 | module4 | module5 | module6 | module7 | module8 | module9 | module10 |
|---|---|---|---|---|---|---|---|---|---|---|---|
| number of gene | 3364 | 1094 | 445 | 328 | 325 | 293 | 272 | 213 | 180 | 141 | 132 |
| Cluster 0 | 32.93 | 9.06 | 31.96 | 62.33 | 10.88 | 40.95 | 11.73 | 4.04 | 21.03 | 3.28 | 6.05 |
| Cluster 1 | 9.21 | 8.68 | 31.8 | 31.12 | 10.51 | 22.6 | 7.58 | 3.13 | 18.36 | 3.23 | 5.09 |
| Cluster 2 | 7.19 | 5.48 | 31.15 | 28.98 | 4.40 | 9.68 | 5.79 | 2.42 | 9.88 | 1.84 | 4.70 |
| Cluster 3 | 5.95 | 4.59 | 15.52 | 27.82 | 3.23 | 8.79 | 3.97 | 1.80 | 6.81 | 1.66 | 4.47 |
| Cluster 4 | 4.15 | 3.87 | 8.46 | 21.51 | 3.21 | 8.69 | 2.47 | 1.76 | 5.73 | 1.34 | 2.49 |
| Cluster 5 | 4.14 | 3.63 | 7.70 | 14.7 | 3.13 | 7.28 | 2.19 | 1.71 | 3.69 | 1.21 | 2.37 |
| Cluster 6 | 3.67 | 3.48 | 6.22 | 7.23 | 3.10 | 6.08 | 2.09 | 1.37 | 3.67 | 1.03 | 2.11 |
| Cluster 7 | 2.94 | 3.30 | 5.02 | 5.44 | 2.99 | 4.72 | 1.65 | 1.32 | 3.03 | 0.90 | 2.05 |
| Cluster 8 | 2.91 | 3.04 | 4.52 | 5.25 | 2.79 | 4.06 | 1.49 | 1.30 | 2.92 | 0.84 | 2.03 |
| Cluster 9 | 2.86 | 2.94 | 4.03 | 4.84 | 2.73 | 3.91 | 1.38 | 1.24 | 2.78 | 0.83 | 1.92 |
| mean score | 7.60 | 4.81 | **14.64** | **20.92** | 4.70 | **11.68** | 4.03 | 2.01 | 7.79 | 1.62 | 3.33 |

| | module11 | module12 | module13 | module14 |
|---|---|---|---|---|
| number of gene | 117 | 113 | 78 | 62 |
| Cluster 0 | 3.94 | 6.94 | 1.88 | 1.03 |
| Cluster 1 | 1.87 | 5.69 | 1.71 | 0.83 |
| Cluster 2 | 1.58 | 5.41 | 1.01 | 0.50 |
| Cluster 3 | 1.44 | 4.32 | 0.87 | 0.16 |
| Cluster 4 | 1.43 | 3.47 | 0.45 | * |
| Cluster 5 | 1.42 | 2.76 | 0.32 | * |
| Cluster 6 | 1.39 | 2.04 | 0.24 | * |
| Cluster 7 | 1.24 | 1.90 | * | * |
| Cluster 8 | 1.21 | 1.27 | * | * |
| Cluster 9 | 1.16 | 0.10 | * | * |
| mean score | 1.67 | 3.39 | 0.93 | 0.63 |

PAAD cancer and KISL

| | module0 | module1 | module2 | module3 | module4 | module5 | module6 | module7 | module8 | module9 | module10 |
|---|---|---|---|---|---|---|---|---|---|---|---|
| number of gene | 440 | 18 | 165 | 719 | 14 | 2234 | 1280 | 21 | 878 | 16 | 396 |
| Cluster 0 | 22.39 | 1.44 | 6.93 | 38.76 | 0.38 | 9.50 | 11.98 | 2.38 | 9.89 | 3.69 | 93.73 |
| Cluster 1 | 11.65 | 1.27 | 6.39 | 12.73 | * | 7.60 | 5.74 | 1.92 | 4.40 | 1.24 | 46.64 |
| Cluster 2 | 6.68 | 0.04 | 4.06 | 10.19 | * | 2.70 | 5.07 | 1.67 | 4.16 | 0.80 | 43.91 |
| Cluster 3 | 4.53 | * | 3.52 | 4.93 | * | 2.37 | 4.95 | * | 3.90 | 0.16 | 33.31 |
| Cluster 4 | 3.49 | * | 3.33 | 4.83 | * | 2.34 | 3.81 | * | 1.96 | * | 24.67 |
| Cluster 5 | 2.85 | * | 2.50 | 4.03 | * | 2.11 | 3.62 | * | 1.95 | * | 15.47 |
| Cluster 6 | 2.45 | * | 2.05 | 3.86 | * | 2.05 | 3.31 | * | 1.57 | * | 13.09 |
| Cluster 7 | 2.42 | * | 1.85 | 3.35 | * | 1.92 | 3.03 | * | 1.47 | * | 9.95 |
| Cluster 8 | 2.37 | * | 1.68 | 3.09 | * | 1.82 | 2.84 | * | 1.41 | * | 5.33 |
| Cluster 9 | 2.35 | * | 1.65 | 3.08 | * | 1.74 | 2.77 | * | 1.38 | * | 5.23 |
| mean score | 6.12 | 0.92 | 3.40 | **8.89** | 0.38 | 3.42 | 4.71 | 1.99 | 3.21 | 1.47 | **29.13** |

| | module11 | module12 | module13 | module14 |
|---|---|---|---|---|
| number of gene | 346 | 83 | 134 | 413 |
| Cluster 0 | 24.38 | 2.40 | 10.23 | 2.01 |
| Cluster 1 | 23.44 | 1.45 | 10.18 | 1.73 |
| Cluster 2 | 22.68 | 1.26 | 5.72 | 1.72 |
| Cluster 3 | 21.23 | 1.12 | 5.32 | 1.67 |
| Cluster 4 | 8.07 | 1.04 | 5.00 | 1.53 |
| Cluster 5 | 6.74 | 1.03 | 3.88 | 1.51 |
| Cluster 6 | 4.83 | 0.79 | 3.11 | 1.50 |
| Cluster 7 | 4.51 | 0.77 | 2.57 | 1.47 |
| Cluster 8 | 4.42 | 0.75 | 2.13 | 1.20 |
| Cluster 9 | 4.38 | 0.26 | 1.98 | 1.05 |
| mean score | **12.47** | 1.09 | 5.01 | 1.54 |

STAD cancer and Pearson-based WGCNA

| | module0 | module1 | module2 | module3 | module4 | module5 | module6 | module7 | module8 | module9 | module10 |
|---|---|---|---|---|---|---|---|---|---|---|---|
| number of gene | 1161 | 1543 | 751 | 668 | 640 | 520 | 454 | 353 | 166 | 154 | 134 |
| Cluster 0 | 54.46 | 28.03 | 6.65 | 6.31 | 2.72 | 2.52 | 1.01 | 2.77 | 78.1 | 9.94 | 5.92 |
| Cluster 1 | 15.46 | 13.46 | 4.49 | 1.80 | 0.64 | 1.95 | 0.85 | 2.45 | 36.69 | 8.91 | 4.44 |
| Cluster 2 | 7.83 | 9.23 | 3.56 | 1.51 | 0.30 | 1.88 | 0.62 | 2.14 | 7.97 | 7.11 | 3.26 |
| Cluster 3 | 6.89 | 6.78 | 3.46 | 1.34 | 0.27 | 1.67 | 0.52 | 2.14 | 6.15 | 5.18 | 3.06 |
| Cluster 4 | 5.95 | 6.16 | 3.33 | 1.31 | 0.25 | 1.25 | 0.31 | 2.11 | 4.26 | 3.45 | 2.74 |
| Cluster 5 | 5.85 | 5.35 | 3.33 | 1.24 | * | 1.12 | 0.30 | 2.07 | 3.57 | 2.74 | 2.50 |
| Cluster 6 | 3.80 | 4.90 | 2.81 | 1.19 | * | 1.08 | * | 1.59 | 3.15 | 2.67 | 2.01 |
| Cluster 7 | 3.65 | 4.62 | 2.49 | 0.92 | * | 1.07 | * | 1.49 | 3.12 | 2.52 | 1.80 |
| Cluster 8 | 3.40 | 3.91 | 2.45 | 0.78 | * | 0.98 | * | 1.48 | 3.08 | 2.40 | 1.63 |
| Cluster 9 | 3.39 | 3.69 | 2.37 | 0.74 | * | 0.89 | * | 1.47 | 2.64 | 2.38 | 1.45 |
| mean score | **11.07** | **8.61** | 3.49 | 1.71 | 0.84 | 1.44 | 0.60 | 1.97 | **14.87** | 4.73 | 2.88 |

| | module11 | module12 | module13 | module14 | module15 | module16 | module17 |
|---|---|---|---|---|---|---|---|
| number of gene | 128 | 92 | 91 | 83 | 66 | 65 | 61 |
| Cluster 0 | 23.61 | 4.71 | 1.73 | 1.95 | 2.44 | 3.53 | 2.81 |
| Cluster 1 | 4.53 | 3.97 | 1.69 | 1.85 | 2.34 | 1.71 | 2.25 |
| Cluster 2 | 4.41 | 2.99 | 1.44 | 1.79 | 1.39 | 1.18 | 2.06 |
| Cluster 3 | 3.38 | 2.76 | 1.20 | 1.31 | 1.27 | 0.66 | 1.95 |
| Cluster 4 | 3.13 | 2.62 | 1.16 | 1.29 | 1.11 | 0.51 | 0.70 |
| Cluster 5 | 2.95 | 1.91 | 1.15 | 0.85 | 0.97 | * | 0.67 |
| Cluster 6 | 2.87 | 0.76 | 0.83 | 0.56 | 0.51 | * | 0.48 |
| Cluster 7 | 2.77 | 0.24 | 0.60 | 0.55 | 0.09 | * | 0.17 |
| Cluster 8 | 2.73 | 0.21 | 0.58 | 0.45 | 0.06 | * | 0.06 |
| Cluster 9 | 2.41 | 0.21 | 0.46 | 0.29 | * | * | * |
| mean score | 5.28 | 2.04 | 1.08 | 1.09 | 1.13 | 1.52 | 1.24 |

STAD cancer and KISL

| | module0 | module1 | module2 | module3 | module4 | module5 | module6 | module7 | module8 | module9 | module10 |
|---|---|---|---|---|---|---|---|---|---|---|---|
| number of gene | 304 | 1173 | 1409 | 143 | 4 | 384 | 439 | 213 | 266 | 454 | 843 |
| Cluster 0 | 3.52 | 28.36 | 21.34 | 2.87 | 2.21 | 4.35 | 2.86 | 2.01 | 54.16 | 2.46 | 2.72 |
| Cluster 1 | 2.86 | 9.91 | 8.96 | 1.75 | * | 1.91 | 2.75 | 1.78 | 51.25 | 2.36 | 0.54 |
| Cluster 2 | 2.72 | 4.77 | 6.65 | 1.21 | * | 1.34 | 2.50 | 1.43 | 25.58 | 2.31 | 0.51 |
| Cluster 3 | 2.59 | 4.72 | 6.46 | 0.48 | * | 1.33 | 2.30 | 1.25 | 23.34 | 1.72 | 0.35 |
| Cluster 4 | 2.07 | 4.00 | 5.52 | 0.39 | * | 1.07 | 2.28 | 1.05 | 17.45 | 1.64 | 0.08 |
| Cluster 5 | 1.90 | 3.36 | 3.97 | 0.34 | * | 1.05 | 2.26 | 0.86 | 11.51 | 1.61 | * |
| Cluster 6 | 1.29 | 3.04 | 3.69 | 0.26 | * | 0.75 | 2.23 | 0.67 | 4.42 | 1.04 | * |
| Cluster 7 | 1.24 | 3.02 | 3.42 | 0.23 | * | 0.58 | 1.88 | 0.53 | 4.40 | 0.96 | * |
| Cluster 8 | 1.10 | 2.95 | 2.81 | 0.17 | * | 0.57 | 1.87 | 0.30 | 4.30 | 0.85 | * |
| Cluster 9 | 0.92 | 2.91 | 2.70 | 0.12 | * | 0.55 | 1.79 | 0.23 | 2.78 | 0.77 | * |
| mean score | 2.02 | 6.70 | 6.55 | 0.78 | 2.21 | 1.35 | 2.27 | 1.01 | **19.92** | 1.57 | 0.84 |

| | module11 | module12 | module13 | module14 | module15 | module16 | module17 |
|---|---|---|---|---|---|---|---|
| number of gene | 468 | 14 | 61 | 86 | 220 | 242 | 407 |
| Cluster 0 | 2.97 | 4.87 | 1.38 | 6.49 | 74.68 | 56.16 | 5.83 |
| Cluster 1 | 2.67 | 0.72 | 1.32 | 4.67 | 11.87 | 25.9 | 3.73 |
| Cluster 2 | 2.48 | 0.06 | 1.14 | 3.65 | 6.82 | 13.38 | 3.60 |
| Cluster 3 | 2.45 | 0.03 | 0.81 | 2.81 | 6.76 | 12.31 | 3.53 |
| Cluster 4 | 1.77 | * | 0.68 | 2.74 | 4.85 | 12.29 | 3.12 |
| Cluster 5 | 1.60 | * | 0.48 | 2.12 | 4.44 | 11.16 | 2.95 |
| Cluster 6 | 1.37 | * | 0.46 | 2.00 | 4.38 | 7.49 | 2.65 |
| Cluster 7 | 1.28 | * | 0.34 | 1.96 | 3.47 | 6.72 | 2.45 |
| Cluster 8 | 1.20 | * | 0.20 | 1.41 | 3.16 | 6.01 | 2.36 |
| Cluster 9 | 1.10 | * | 0.20 | 1.19 | 2.89 | 5.23 | 2.19 |
| mean score | 1.89 | 1.42 | 0.70 | 2.90 | **12.33** | **15.66** | 3.24 |
