# Supplementary material for: KISL: knowledge-injected semi-supervised learning for biological co-expression network modules
Source: Front Genet. 2023 May 2;14:1151962. doi: 10.3389/fgene.2023.1151962 (PMC10185879; doi:10.3389/fgene.2023.1151962)
Supplement: Supplementary file 2 [file Table4.pdf]

Table S4: Clustering evaluation score in each dataset

		BLCA	BRCA	COAD	KIRC	LUAD	LUSC	STAD	PAAD
SI	WGCNA	-0.0157	-0.0506	-0.0242	-0.0225	-0.1714	0.04	-0.0232	-0.0051
	KISL	0.08	0.177	0.209	0.1248	0.1778	0.2912	0.1323	0.124
CH	WGCNA	341.08	470.26	1011.14	267.65	392.85	1489.54	572.56	490.41
	KISL	1019.08	3869.98	3562.82	1118.46	3743.60	9358.68	1211.30	1404.33
DBI	WGCNA	5.18	3.74	3.53	3.90	4.59	3.67	4.29	5.60
	KISL	2.32	1.48	1.19	1.88	1.30	0.99	1.60	1.84
Maximum module density	WGCNA	0.1527	0.1384	0.2935	0.4710	0.2531	0.3358	0.1903	0.2533
	KISL	0.4047	0.4573	0.4237	0.6180	0.6531	0.5141	0.5704	0.4801
