# Supplementary material for: KISL: knowledge-injected semi-supervised learning for biological co-expression network modules
Source: Front Genet. 2023 May 2;14:1151962. doi: 10.3389/fgene.2023.1151962 (PMC10185879; doi:10.3389/fgene.2023.1151962)
Supplement: Supplementary file 4 [file Table2.pdf]

Table S2

Datasets	BLCA	BRCA	COAD	KIRC	LUAD	LUSC	PAAD	STAD
Loss	29.41	43.18	49.07	33.18	61.04	66.37	70.89	50.62
