# Supplementary material for: KISL: knowledge-injected semi-supervised learning for biological co-expression network modules
Source: Front Genet. 2023 May 2;14:1151962. doi: 10.3389/fgene.2023.1151962 (PMC10185879; doi:10.3389/fgene.2023.1151962)
Supplement: Supplementary file 5 [file Table3.pdf]

Table S3: summary of pairwise constraints

	BLCA	BRCA	COAD	KIRC	LUAD	LUSC	STAD	PAAD
Number of gene	6253	7138	6723	5291	6749	8748	7130	7157
Number of gene pair	1692	1791	3791	1670	1918	2187	1970	1423
ratio of constrained gene	0.1091	0.108	0.1342	0.1227	0.1073	0.0918	0.1128	0.0896
