# Supplementary material for: KISL: knowledge-injected semi-supervised learning for biological co-expression network modules
Source: Front Genet. 2023 May 2;14:1151962. doi: 10.3389/fgene.2023.1151962 (PMC10185879; doi:10.3389/fgene.2023.1151962)
Supplement: Supplementary file 6 [file Image1.pdf]

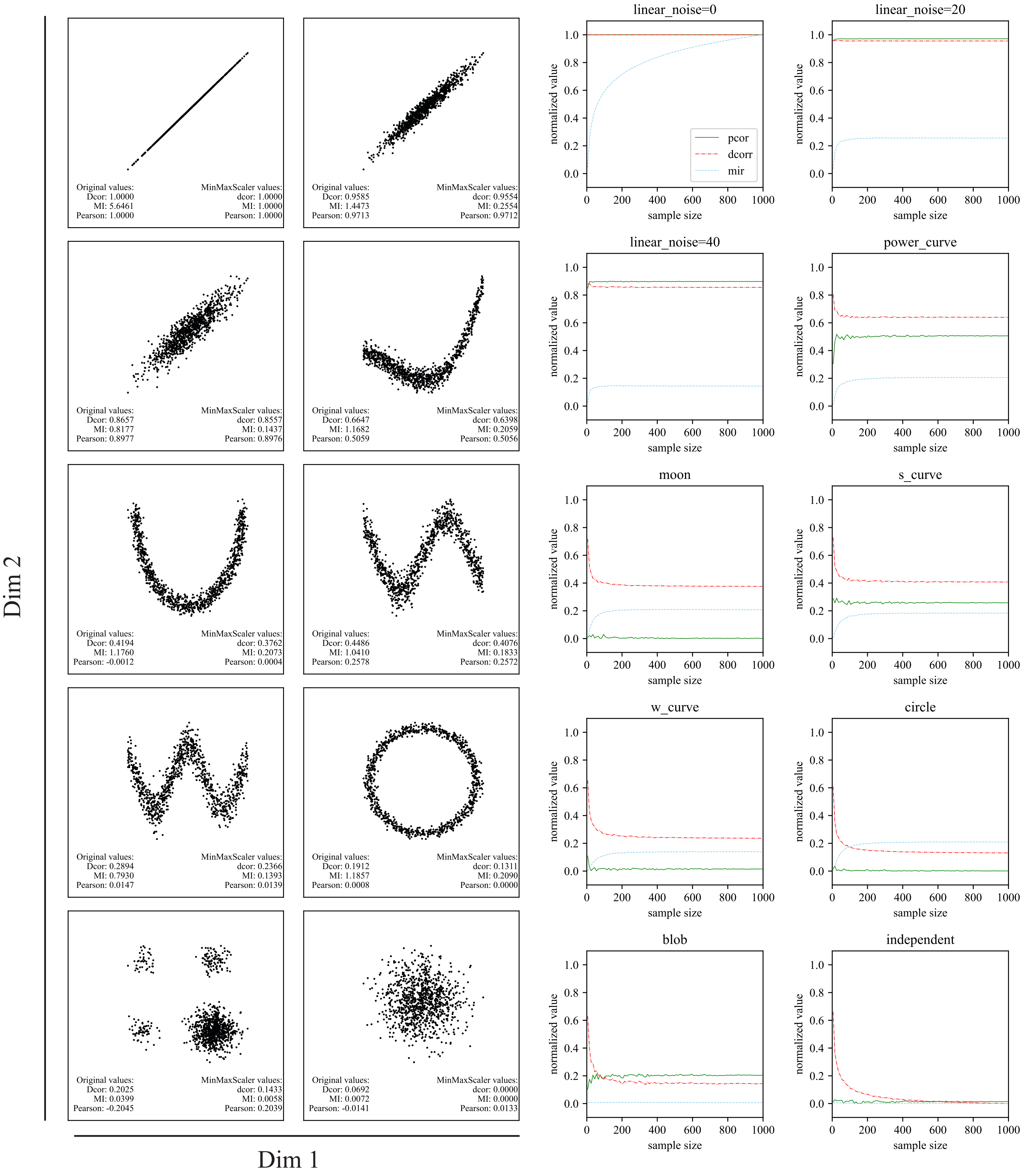

Figure S1 According to the figure, distance correlation captures complex relationships best. Additionally, mutual information can capture complex relationships, especially those involving circular dependencies. The Pearson correlation coefficient is the linear part that only captures dependence. Distance correlation coefficient and Pearson correlation coefficient have more obvious capture abilities among variables with strong linear dependence and exponential dependence. Furthermore, we can see from the completely linear dependence data that the sample size will affect mutual information. Perhaps this is due to the fact that information entropy can only be calculated from discrete data, and sampling results in information loss. Furthermore, mutual information has the strongest anti-noise properties, and the increase in noise will reduce the distance correlation coefficient. The complex relationship between low-latitude variables can be difficult to capture using mutual information and distance correlation. Overall, the distance correlation has some advantages in capturing linear and nonlinear dependencies between variables.
